# Evaluation of a Question Prompt List About Cardiovascular Disease Risk and Prevention After Hypertensive Pregnancy: A Pilot Study

**DOI:** 10.1111/hex.70085

**Published:** 2024-10-30

**Authors:** Smita Dhakal, Bethany Rankin, Taryn Assaf, Jane Baker, Laura Chisick, Tracey Colella, Natalie Dayan, Maureen Dobbins, Sherry Grace, Serena Gundy, Sheila O'Keefe McCarthy, Ziran Meng, Beth Murray‐Davis, Sarah Neil‐Sztramko, Kara Nerenberg, Winnie Sia, Graeme Smith, Maria Timofeeva, Anna R. Gagliardi

**Affiliations:** ^1^ Toronto General Hospital Research Institute University Health Network Toronto Ontario Canada; ^2^ Briar Hill Midwives Calgary Alberta Canada; ^3^ Allin Clinic University of Alberta Edmonton Alberta Canada; ^4^ Health Science Centre University of Manitoba Winnipeg Manitoba Canada; ^5^ Cardiovascular Prevention and Rehabilitation Program University Health Network Toronto Ontario Canada; ^6^ McGill University Health Centre McGill University Montreal Quebec Canada; ^7^ Department of Health Research Methods, Evidence, and Impact McMaster University Hamilton Ontario Canada; ^8^ Faculty of Health York University Toronto Ontario Canada; ^9^ McMaster University Medical Centre McMaster University Hamilton Ontario Canada; ^10^ Department of Nursing Brock University Guelph Ontario Canada; ^11^ Women's Heart Clinic Queen Elizabeth II Hospital Halifax Nova Scotia Canada; ^12^ Midwifery Education Program McMaster University Hamilton Ontario Canada; ^13^ Foothill Medical Centre University of Calgary Calgary Alberta Canada; ^14^ Royal Alexandra Hospital University of Alberta Edmonton Alberta Canada; ^15^ Maternal Health Clinic Kingston General Hospital Kingston Ontario Canada; ^16^ Department of Cardiology Women's College Hospital Toronto Ontario Canada

**Keywords:** cardiovascular disease, communication, hypertensive disorders of pregnancy, qualitative interview, risk reduction, question prompt list

## Abstract

**Introduction:**

The aim of this study was to pilot test a question prompt list (QPL) about cardiovascular disease (CVD) risk reduction after hypertensive pregnancy (HDP).

**Methods:**

In a prospective cohort study of adult women who had HDP given the QPL before and surveyed after a physician visit, we assessed perceived person‐centred care, self‐efficacy for self‐management, perceived self‐management and QPL feasibility.

**Results:**

Twenty‐three women participated: 57% of diverse ethno‐cultural groups, 65% < 40 years of age and 48% immigrants. Most scored high for person‐centred care (mean 4.1 ± 0.2/5); and moderately for self‐efficacy (mean 7.4 ± 0.6/10) and self‐management (mean 3.1 ± 0.3/5). Most appreciated QPL design and reported QPL benefits: helped them to prepare for the visit and know what to ask; increased confidence to ask questions, knowledge of the link between HDP and CVD and lifestyle behaviours to reduce CVD risk. Most reported that physicians were receptive to discussing QPL questions.

**Conclusion:**

Women appreciated the QPL and knowledge about self‐management was high but self‐efficacy for or perceived self‐management was moderate. It appears feasible to share a QPL with ethno‐culturally diverse women who can share it with physicians to facilitate discussions about post‐pregnancy HDP‐related CVD risk.

**Patient or Public Contribution:**

This study involved women who experienced HDP and engaged ethno‐culturally diverse women with lived experience of HDP as study advisors in all stages of the research.

**Abbreviations**
CPCN: Canadian Post‐pregnancy Clinical Network
CVD: cardiovascular disease
HDP: hypertensive disorders of pregnancy
PCC: person‐centred care
QPL: question prompt list

## 1 Introduction

Hypertensive disorders of pregnancy (HDP), a spectrum of conditions from gestational hypertension to severe pre‐eclampsia with or without eclampsia, affects up to 10% of all pregnancies [1, 2]. HDP heightens the risk of future premature cardiovascular disease (CVD) by two to five times compared to women with normotensive pregnancies [3, 4, 5]. CVD warning signs typically appear within 5 years, and CVD events within 10 years postpartum [1]. Women with a first pregnancy at age ≥ 40 years, with obesity, without a previous diagnosis of hypertension and/or of certain ethno‐cultural groups (non‐Hispanic Black, Filipino and South Asian) have a higher risk of post‐HDP CVD [5, 6, 7]. Guidelines recommend preventing HDP‐related CVD through physical activity, healthy diet, weight loss, pharmacotherapy and counselling women on CVD risk reduction [5, 8, 9, 10, 11, 12].

However, research in Canada [13], Egypt [14], Germany [15], Portugal [16], the United Kingdom [17] and the United States [2, 18, 19, 20, 21] revealed that women with HDP were not alerted about CVD risk or how to prevent it. In those studies, women described a lack of person‐centred care: physicians said they were over‐reacting about normal postpartum symptoms or attributed CVD symptoms to migraines, flu, indigestion, anxiety, asthma or constipation [13, 22, 23]. Women felt dismissed, disrespected and distressed about their health; and wanted more information, but avoided asking questions in case physicians perceived them as difficult [13, 19, 20, 22]. A review of 16 studies involving women with HDP from higher‐ and lower‐income countries reported similar findings [24]. Physicians lack knowledge of HDP: 50% of Canadian family physicians, cardiologists and obstetricians surveyed in 2007 [25] and 2018 [26] were unaware of HDP‐related CVD risk and prevention, a finding confirmed by a review of six studies involving physicians from Australia, Canada, Germany, Nigeria and the United States [21].

We lack insight into person‐centred strategies to raise awareness about CVD prevention among affected women and physicians. A review of interventions to mitigate post‐HDP CVD risk identified only eight studies, all involving counselling targeting women only, of which none improved CVD risk factors [27]. Research points to the need for tools that support patient–provider discussion about HDP‐related CVD risk and prevention. Australian midwives, general practitioners, obstetrician‐gynaecologists and cardiologists said that they wanted printed or online material to facilitate HDP discussions [28, 29]. Similarly, Australian [30] and American [19] women with HDP recommended a discussion guide to help them ask physicians about CVD risk and prevention.

Question prompt lists (QPLs) on various topics improved patient question‐asking, satisfaction with communication and the amount and quality of information provided by physicians without increasing consultation length [31, 32, 33, 34, 35]. Since women with HDP felt dismissed and lacked the confidence to ask questions [13, 19, 20, 22] and women and physicians requested communication aids [19, 28, 29, 30], a QPL about HDP could encourage person‐centred exchange about HDP‐related CVD risk and prevention by empowering women to articulate questions and priming physicians to address concerns. Such discussions may prompt preventive lifestyle behaviour among women, thereby reducing CVD risk. This approach is supported by prior research showing that communication tools targeting both patients and physicians are more likely to be used compared with targeting only patients [36], as was done in all prior interventions to mitigate post‐HDP CVD risk [27]. The overall aim of this research was to pilot test a QPL about HDP in preparation for a future trial of QPL use and impact. The objectives were to assess the potential impact of the QPL on perceived person‐centred care (PCC), and confidence to self‐manage CVD prevention and the feasibility of QPL uptake.

## 2 Methods

### 2.1 Approach

We employed a multiple‐methods research design to pilot‐test a QPL on HDP [37, 38]. In a prospective cohort study, we shared a QPL about HDP with women before a physician visit, and assessed outcomes among women after the visit. We focused directly on QPL uptake by women, and indirectly on uptake by physicians based on women's reports of physician response. We complied with reporting criteria for observational and qualitative studies [39, 40]. The research team included four women who had HDP as research advisors; eight health services researchers with expertise in implementation science, nursing, midwifery, cardiac rehabilitation and obstetric internist; and seven obstetricians or obstetric internists who helped to recruit women.

### 2.2 QPL for HDP

We complied with the Framework for Design and Evaluation of Complex Interventions [41]. In Stage 1, we synthesised research on the optimal design and implementation of QPLs [33] and interviewed 22 women who had HDP from across Canada to identify QPL questions [42]. In Stage 2, we shared the draft QPL with, and interviewed 21 diverse women and 21 physicians about QPL design, use and impact and used that knowledge to refine QPL and study design [13]. Stage 3 of the Framework comprises this pilot test in preparation for Stage 4, a future trial. The QPL is available at https://arglab.ca/wp-content/uploads/2023/01/HDP-QPT.pdf.

### 2.3 Sampling and Recruitment

Eligible women were aged 18+ who had at least one HDP pregnancy. Using maximum variation sampling, we aimed to recruit 24 women including those at higher risk of HDP‐related CVD (African or Caribbean Black or South Asian), who also varied by age, region of Canada and immigration status [43]. We identified and recruited women using two approaches. To simulate reaching women via physicians, we asked physicians at seven interested Canadian Post‐pregnancy Clinical Network (CPCN) sites to identify newly referred eligible women and provide them with a study information sheet. The CPCN includes 20+ clinics across Canada focused on vascular risk reduction after HDP [44]. We met with physicians in advance of the study launch and provided them with a 7‐min narrated slide show, reading list and two versions of the QPL, one with and one without answers (available at https://arglab.ca/hdp/). To simulate reaching women directly, managers of immigrant settlement agencies and prenatal classes and midwives contacted by the Canadian Association of Midwives shared the information sheet with women clients. We also asked participating women to share the information sheet with family, friends or colleagues who had HDP. The information sheet invited interested eligible women to contact the study coordinator, who established informed consent. Once consented, we sent women a reading list of credible sites offering information about HDP and preventing or managing CVD, the aforementioned QPL and a 4‐min narrated slide show describing how to use the QPL before and during their next physician visit (available at https://arglab.ca/hdp/). Recruitment was launched on 3 January 2023 and concluded on 28 March 2024.

### 2.4 Data Collection

Within 2 weeks of physician visits before or during which women may have read or referred to the QPL, we conducted telephone interviews with women to collect self‐reported demographic information, administer instruments and ask qualitative questions. We entered instrument answers into an Excel spreadsheet, and the qualitative portion was audio‐recorded and transcribed. To assess perceived PCC during the physician visit, we employed the Consultation Care Measure (CCM), a validated, reliable instrument of 21 items in six domains rated on a 5‐point Likert scale from strongly disagree to strongly agree [45]. We employed two instruments to assess confidence and perceived self‐management. The Self‐efficacy for Managing Chronic Disease Scale (SMCDS) is a validated, reliable instrument of 6 items scored on a 10‐point scale where 10 represents the highest agreement [46]. The Perceived Medical Condition Self‐Management Scale (PMCSMS) is a validated, reliable instrument of eight items scored on a 5‐point Likert scale from strongly disagree to strongly agree [47]. To assess the feasibility of QPL uptake, we asked women qualitative questions (Supporting Information: File 1) about how they used the QPL before and during a physician visit, how the physician reacted, QPL impact and enablers and barriers of QPL use.

### 2.5 Data Analysis

We used summary statistics calculated in Excel to describe participant characteristics and instrument scores. We calculated response frequencies for each instrument item and mean score, total mean score and standard deviation of mean score for instrument items and participants. In keeping with the qualitative description, we used content analysis to identify themes inductively through constant comparison and used Excel and Word to manage data [38]. Two researchers: The principal investigator and a research associate independently coded transcripts of the first two interviews, and then met to discuss and refine a preliminary codebook of themes and exemplar quotes. Thereafter, the research associate coded all transcripts and expanded or merged themes as needed. ARG independently reviewed all coding. We tabulated data (themes and quotes) to compare themes between women with diverse demographic characteristics, and those recruited by physicians versus other means.

## 3 Results

### 3.1 Participants

**Figure 1:**
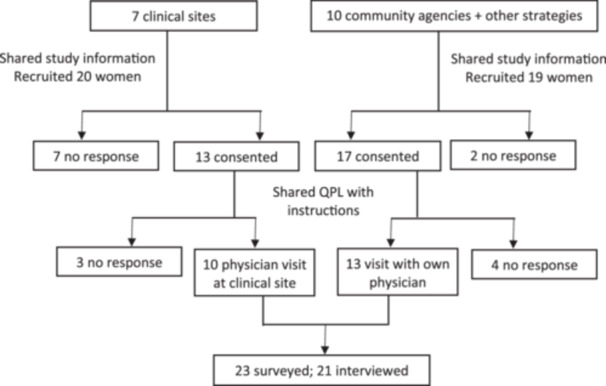
Participation flow diagram. This figure shows the participant recruitment and retention process, capturing responses, consent, non‐responses post‐consent and the final sample of 23 surveyed participants, including 21 who were interviewed.

Figure 1 depicts the number of women who consented and ultimately participated. Ten women were recruited by seven physicians, and 13 women via 10 community agencies or other means for a total of 23 participants. Most (13, 56.5%) participants represented diverse ethno‐cultural groups, were under the age of 40 (15, 65.2%) and lived in the Ontario region (18, 78.3%). Nearly half (11, 47.8%) were immigrants to Canada (Table 1). The mean time from women's receipt of the QPL to physician visit was 12.3 days (median 12, range 1–32). The majority of women saw a family physician (15, 65.2%) followed by an obstetrician and gynaecologist (6, 26.1%) and 1 (4.3%) each a general internal medicine physician and obstetric internist.

**Table 1:** Characteristics of participating women.

Characteristic	*n* (% of 23)
Ethno‐cultural group	
Caucasian	10 (43.5)
African Black	8 (34.8)
South Asian	4 (17.4)
Indigenous	1 (4.3)
Age (years)	
20–29	4 (17.4)
30–39	11 (47.8)
40–49	7 (30.4)
50+	1 (4.3)
Province	
Alberta	3 (13.0)
Ontario	18 (78.3)
Quebec	2 (9.0)
Years in Canada	
Born in Canada	12 (52.2)
< 10 years	5 (21.7)
10+ years	6 (26.1)
Recruitment	
Physicians	10 (43.5)
Community agencies/other strategies	13 (56.5)

### 3.2 Person‐Centred Care

Supporting Information: File 2 provides CCM participant and item scores. Table 2 summarises item scores. Findings did not differ by women's characteristics or recruitment method. The overall participant mean score was 4.1 (± 0.2/5.0, range 2.7–5.0), indicating that most individuals similarly perceived high PCC. The overall item mean score was 4.1 (± 0.6/5.0, range 3.5–4.4), indicating that most items across PCC domains similarly scored high. Items that scored the lowest were ‘The doctor was interested in what treatment I wanted’ in the Communication and Partnership domain (mean 3.5 ± 0.9) and both items (‘The doctor showed interest in the effect of the problem on me, and family/personal life’; ‘The doctor showed interest in the effect of the problem on my daily activities’) in the Interest in Life Impact domain (mean 3.7; ± 1.2 and 1.0, respectively).

**Table 2:** Summary of consultation care measure scores for perceived person‐centred care.

Item	Domain	Statement	Agreement with item *n* (% of 23 participants)					Overall item score	Overall item mean^a^ (SD)
			Strongly disagree	Disagree	Neutral	Agree	Strongly agree		
1	Relationship	The doctor knows me and understands me or asks questions to get to know me and understand me	1 (4.3)	1 (4.3)	3 (13.0)	8 (34.8)	10 (43.5)	94	4.1 (1.1)
2		The doctor understands my emotional needs or asks questions to assess my emotional needs	1 (4.3)	2(8.7)	2 (8.7)	10 (43.5)	8 (34.8)	91	4.0 (1.1)
3		I'm confident that the doctor knows me and my medical history	1 (4.3)	0 (0.0)	3 (13.0)	11 (47.8)	8 (34.8)	94	4.1 (0.9)
4	Communication and partnership	The doctor was interested to know about my worries about my health	0 (0.0)	1 (4.3)	3 (13.0)	11 (47.8)	8 (34.8)	95	4.1 (0.8)
5		The doctor was interested when I talked about my symptoms	0 (0.0)	1 (4.3)	1 (4.3)	11 (47.8)	10 (43.5)	99	4.3 (0.8)
6		The doctor was interested in what I wanted to know	0 (0.0)	1 (4.3)	1 (4.3)	10 (43.5)	11 (47.8)	100	4.3 (0.8)
7		I felt encouraged to ask questions	1 (4.3)	1 (4.3)	0 (0.0)	7 (30.4)	14 (60.9)	101	4.4 (1.0)
8		The doctor was careful to explain the plan of treatment	0 (0.0)	1 (4.3)	1 (4.3)	12 (52.2)	9 (39.1)	98	4.3 (0.8)
9		The doctor was sympathetic	0 (0.0)	0 (0.0)	4 (17.4)	9 (39.1)	10 (43.5)	98	4.3 (0.8)
10		The doctor was interested in what I thought the problem was	0 (0.0)	1 (4.3)	2 (8.7)	12 (52.2)	8 (34.8)	96	4.2 (0.8)
11		The doctor discussed and agreed together with me what the problem was	0 (0.0)	1 (4.3)	3 (13.0)	10 (43.5)	9 (39.1)	96	4.2 (0.8)
12		The doctor was interested in what I wanted	0 (0.0)	1 (4.3)	2 (8.7)	10 (43.5)	10 (43.5)	98	4.3 (0.8)
13		The doctor was interested in what treatment I wanted	0 (0.0)	2 (8.7)	12 (52.2)	4 (17.4)	5 (21.7)	81	3.5 (0.9)
14		The doctor discussed and agreed together with me on the plan of treatment	0 (0.0)	1 (4.3)	4 (17.4)	12 (52.2)	6 (26.0)	92	4.0 (0.8)
15	Health promotion	The doctor shared ideas on ways to lower the risk of future illness	1 (4.3)	0 (0.0)	2 (8.7)	8 (34.8)	12 (52.2)	99	4.3 (1.0)
16		The doctor advised me on how to prevent future health problems	0 (0.0)	1 (4.3)	1 (4.3)	11 (47.8)	10 (43.5)	99	4.3 (0.8)
17	Positive and clear approach to problem	The doctor explained clearly what the problem was	0 (0.0)	1 (4.3)	2 (8.7)	11 (47.8)	9 (39.1)	97	4.2 (0.8)
18		The doctor was sure about what the problem was	0 (0.0)	2 (8.7)	4 (17.4)	7 (30.4)	10 (43.5)	94	4.1 (1.0)
19		The doctor was sure about when or how the problem could be managed	0 (0.0)	1 (4.3)	6 (26.0)	6 (26.0)	10 (43.5)	94	4.1 (0.9)
20	Interest in life impact	The doctor showed interest in the effect of the problem on me, and family/personal life	0 (0.0)	5 (21.7)	4 (17.4)	6 (26.0)	8 (34.8)	86	3.7 (1.2)
21		The doctor showed interest in the effect of the problem on my daily activities	0 (0.0)	3 (13.0)	5 (21.7)	10 (43.5)	5 (21.7)	86	3.7 (1.0)

Abbreviation: SD, standard deviation.

^a^ /5, with higher scores more positive.

### 3.3 Self‐Management

Supporting Information: File 3 provides SMCDS participant and item scores. Table 3 summarises item scores. Findings did not differ by women's characteristics or recruitment method. The overall participant mean score was 7.4 (± 0.6/10.0, range 5.0–9.5), indicating that most individuals similarly scored moderately for self‐efficacy for self‐management. The overall item mean score was 7.4 (± 0.5/10.0, range 6.8–8.1), indicating that most items similarly scored moderately for self‐efficacy. Items that scored the lowest were “How confident are you that you can keep the symptoms of HDP or heart disease from interfering with things you want to do?” (mean 6.9 ± 1.8) and “How confident are you that you can keep the emotional distress (feelings like anxiety, depression or frustration) caused by HDP or heart disease from interfering with the things you want to do?” (mean 6.8 ± 1.9).

**Table 3:** Summary of self‐efficacy for managing chronic disease scores.

Item	Statement	Agreement with items on 10‐point scale where 10 is the highest agreement n (% of 23 participants)										Overall item score	Overall item mean^a^ (SD)
		1	2	3	4	5	6	7	8	9	10		
1	How confident are you that you can keep the symptoms of HDP or heart disease from interfering with things you want to do?	0 (0.0)	0 (0.0)	1 (4.3)	0 (0.0)	6 (26.9)	2 (8.7)	4 (17.4)	6 (26.9)	2 (8.7)	2 (8.7)	159	6.9 (1.8)
2	How confident are you that you can keep any physical discomfort, pain and/or other symptoms caused by HDP or heart disease from interfering with the things you want to do?	0 (0.0)	0 (0.0)	0 (0.0)	1 (4.3)	3 (13.0)	5 (21.7)	4 (17.4)	6 (26.9)	3 (13.0)	1 (4.3)	162	7.0 (1.6)
3	How confident are you that you can keep the emotional distress (feelings like anxiety, depression or frustration) caused by HDP or heart disease from interfering with the things you want to do?	0 (0.0)	1 (4.3)	0 (0.0)	2 (8.7)	2 (8.7)	3 (13.0)	6 (26.9)	5 (21.7)	3 (13.0)	1 (4.3)	157	6.8 (1.9)
4	How confident are you that you can keep any other symptoms or health problems you have* other than HDP or heart disease* from interfering with the things you want to do?	0 (0.0)	1 (4.3)	0 (0.0)	0 (0.0)	0 (0.0)	3 (13.0)	5 (21.7)	7 (30.4)	5 (21.7)	2 (8.7)	176	7.7 (1.7)
5	How confident are you that you can do recommended things (exercise, healthy eating) to manage your health to reduce your need to see a doctor?	0 (0.0)	0 (0.0)	0 (0.0)	1 (4.3)	1 (4.3)	1 (4.3)	3 (13.0)	7 (30.4)	5 (21.7)	4 (17.4)	186	8.1 (1.6)
6	How confident are you that you can do things other than just taking medication to reduce how much HDP or heart disease affects your everyday life?	0 (0.0)	0 (0.0)	0 (0.0)	1 (4.3)	1 (4.3)	1 (4.3)	4 (17.4)	9 (39.1)	7 (30.4)	0 (0.0)	178	7.7 (1.3)

Abbreviation: SD, standard deviation.

^a^ /10, with higher scores more positive.

Supporting Information: File 4 provides PMCSMS individual and item scores. Table 4 summarises item scores. Findings did not differ by women's characteristics or recruitment method. The overall participant mean score was 3.1 (± 0.3/5.0, range 2.5–3.6), indicating that most individuals similarly scored moderately for self‐management. The overall item mean score was 3.1 (± 0.6/5.0, range 2.4–3.9), indicating that most items similarly scored moderately for self‐management. Items that scored the lowest were “Most of the time, the things I do to prevent or manage heart disease do not work out well” (mean 2.4 ± 0.7) and “No matter how hard I try, things I do to prevent or manage heart disease do not turn out the way I would like” (mean 2.4 ± 0.7).

**Table 4:** Summary of perceived medical condition self‐management scale scores.

Item	Item statement	Agreement with item *n* (% of 23 participants)					Total item score	Item mean^a^ (SD)
		Strongly disagree	Disagree	Neutral	Agree	Strongly agree		
1	It is difficult for me to find good ways to deal with problems that occur when I try to prevent or manage heart disease	0 (0.0)	11 (47.8)	5 (21.7)	7 (30.4)	0 (0.0)	65	2.8 (0.9)
2	I find that when I try to make changes to prevent or manage heart disease, they do not work well	1 (4.3)	11 (47.8)	6 (26.0)	5 (21.7)	0 (0.0)	61	2.7 (0.9)
3	I do things well to prevent or manage heart disease	0 (0.0)	3 (13.0)	6 (26.0)	12 (52.2)	2 (8.7)	82	3.6 (0.8)
4	I am able to do things to prevent or manage heart disease as well as most other people	0 (0.0)	0 (0.0)	6 (26.0)	14 (60.9)	3 (13.0)	89	3.9 (0.6)
5	I succeed in things I do to prevent or manage heart disease	0 (0.0)	3 (13.0)	3 (13.0)	14 (60.9)	3 (13.0)	86	3.7 (0.9)
6	Most of the time, the things I do to prevent or manage heart disease do not work out well	1 (4.3)	13 (56.5)	7 (30.4)	2 (8.7)	0 (0.0)	56	2.4 (0.7)
7	No matter how hard I try, things I do to prevent or manage heart disease do not turn out the way I would like	1 (4.3)	14 (60.9)	6 (26.0)	2 (8.7)	0 (0.0)	55	2.4 (0.7)
8	Most of the time, I am able to reach the goals I set to prevent or manage heart disease	0 (0.0)	2 (8.7)	7 (30.4)	13 (56.5)	1 (4.3)	82	3.6 (0.7)

Abbreviation: SD, standard deviation.

^a^ /5, with higher scores more positive.

### 3.4 Feasibility

Supporting Information: File 5 provides all themes and quotes from interviews with participating women. Table 5 summarises key themes with exemplar quotes that include participant number, province of residence, ethno‐cultural group and age. The majority of women described similar themes regardless of demographic characteristics or recruitment method.

**Table 5:** Summary of themes about QPL feasibility and impact.

Feasibility of QPL		
Interview question	Theme	Exemplar quote (participant number and characteristics)
QPL use before physician visit	Most women printed the QPL to bring to appointment	I printed it and I put it into my purse so it was ready to go for the appointment (15 ON South Asian aged 41)
	Some women saved QPL on phone to bring to appointment	I did not print it. I made a note on my phone (16 ON African aged 29)
	Many women reviewed questions in advance to decide which to ask	I read all the questions. Then I said okay, those are good questions that I should discuss with her (13 QC African aged 42)
QPL use during physician visit	Some women referred to QPL to ask questions but did not show it to the physician	Oh I didn't show it to my doctor. I just told him that I had questions and kept going back to my phone and asking him again or asking a different question… I just felt like a little shy because I don't want to let the doctor know that maybe he's not telling me everything he's supposed to (02 ON Caucasian aged 45)
	Most women felt comfortable showing the QPL to physicians and asking questions	My doctor is very comfortable to discuss all these things. So I can freely speak to her about any issues. I just showed the [QPL] to my doctor and…I just want to know more about that thing (07 ON South Asian aged 40)
Physician reaction to QPL	Most women said the physician was open to jointly review and discuss QPL question	He was so pleased about it because he was like, you are trying to know more about your body and also to take concern about your life. And he was so much happy I'm investigating more about the HDP (17 ON African aged 30)
		We discussed answers to every single question (15 ON South Asian aged 41)
	Some women said the physician asked additional questions or provided more information	It prompted my doctor to use that Framingham calculator…they were able to calculate a risk of heart disease for me over the next 10 years (06 ON Caucasian aged 36)
	A few women said the physician provided brief or dismissive answers or refused to answer questions	Most of like my family history has heart disease or hypertension. I shared that with him but he didn't discuss how that might affect me and things that I can actually do about it. I found, when I asked questions it was, he gave very short answers (22 ON Indigenous aged 26)
		My doctor say that I have no time to take part in such activities and because I'm a little bit busy, I have schedule, like appointments. So I don't, like I am not interested. That's the main problem, that I couldn't get answer from my doctor (08 ON South Asian aged 31)
Enablers of QPL use	Many women said that getting QPL in advance helped them prepare for the appointment	Having it before was really helpful because…you can look at the resources to understand hypertension, so you kind of understand a little bit about the questions that you would want to ask and discuss at the appointment instead of just going into the appointment completely blind (05 ON Caucasian aged 35)
	Some women said the physician was receptive to discussion of QPL questions	It was easy, it was easy. She took all the time with me and then we go over it. I didn't have any, any problem using it, not at all. (13 QC African aged 42)
	A few women mentioned print and electronic formats available to suit different preferences	I just downloaded it onto my phone so that made it really easy for me to bring in with me (02 ON Canadian aged 45)
	A few women noted QPL was well designed (not too many questions, space for answers)	I would say it was brief and it was very well detailed. The questions were not a lot and the questions were straight (09 ON African aged 32)
Barriers to QPL use	Many women said they did not encounter any barriers	Nothing made it hard. It was very easy (15 ON South Asian aged 41)
	A few women said the physician was in a rush and dismissed their concerns	It's just that he's always in a rush. I always feel like when I go to the clinic he's in a rush If I go, something is bugging me, he just goes, oh its nothing. To me it shows like he's shutting you down instead of allowing you to actually express what you're worries are. For most of my issues, I find he brushes things aside very easily (14 ON African aged 46).

Impact of QPL		
Theme	Sub‐theme	Exemplar quote
Prepared women for physician visit	Most women said QPL helped them know what to ask	So I had the idea what to ask (10 AB South Asian aged 46)
	A few women said the QPL helped them initiate a conversation	It got the conversation started so I thought that was very effective. Without the QPL we wouldn't even have the conversation started or any preventative actions discussed (03 AB Caucasian aged 36)
	Most women said QPL increased confidence to self‐advocate	It gave me a foundation to think about my own situation and questions that I should ask and it did ease some anxiety about that appointment (22 ON Indigenous aged 26)
Knowledge about the link between HDP and cardiovascular disease	Most women said they felt confident and motivated to self‐manage their health based on this new‐found knowledge	It definitely gave me a more in depth idea of why this happens in women who had high blood pressure in pregnancy. I definitely feel like I know a lot more of the warning signs and how to prevent it (23 ON Caucasian aged 30)
Knowledge about healthy lifestyle behaviours	Most women said the physician encouraged physical activity	She recommend me to do the exercise. She told me that join yoga class or walk or any exercise (08 ON South Asian aged 31)
	Most women said the physician recommended a healthy diet	Eating well and avoiding a lot of processed food and things that's high in cholesterol and stuff like that (16 ON African aged 29)
	A few women said the physician advised getting enough sleep	Try to have good sleep, try to have at least 6, 7 h of sleep (13 QC African aged 42)
	A few women said the physician advocated stress management	I don't need to have stress or anxieties. I have to be keep and calm, I have to make myself happier (07 ON South Asian aged 40)
	A few women said the physician suggested controlling cholesterol through diet, exercise and medication	I have cholesterol level that could be medicated, but he thinks it can be controlled with diet and exercise, and he was gonna send me a letter explaining a treatment plan (04 ON Caucasian aged 40)
	A few women said the physician recommended the following: – Monitor blood pressure at home – Maintain healthy body weight – Avoid smoking – Avoid alcohol use	They told me to use my at home blood pressure monitor more and keep recordings of my results. They told me when it gets to a certain point consistently to call them and discuss further treatment (06 ON Caucasian aged 36)

Suggested improvements to QPL		
Theme	Sub‐theme	Exemplar quote
Content	Most women said no changes were needed to the content	I think your presentation is quite good, because most of them are not too shareable, but yours is actually very readable and very presentable, so I have no questions on that (19 ON African aged 30)
Format	A few women suggested the format be modifiable (e.g. converted to checklist, able to add questions or discussion points)	If it only can be one sheet, maybe to have a place for extra questions that maybe don't fall into these pre‐set questions (06 ON Caucasian aged 36)
	A few women suggested enhancing the graphic appeal	Have some graphics so it doesn't look like a boring brochure. Maybe an image of a woman and welcoming colours so it looks like something you would want to peek and read (16 ON African aged 29)

Disseminating and supporting QPL use		
Theme		Exemplar quote
Many women said to ensure physicians are familiar with QPL		I think that, like doctors should probably have it as well and kind of use it as like a foundation for appointments for new patients especially (22 ON Indigenous aged 26)
A few women said to reach pregnant women through clinics and pre‐natal classes		Anytime we have a chance with pregnant women or post‐partum women…if there are clinics that receiving pregnant women, I think that we should disseminate the QPL like early, maybe at the end of the pregnancy, mid‐pregnancy (13 QC African aged 42)
Most women said to share the QPL with women rather than being shared by physician during visit		If they only get it at their doctor's office, they may not have time to think of other questions (06 ON Caucasian aged 36)

Abbreviation: QPL, question prompt list.

Most women printed the QPL to take with them to a physician visit and read the QPL in advance to decide which questions they wanted to ask physicians.

I just went through the question and I just ticked on some of the question that I'm gonna be asking

(18 ON African aged 29)

Most women felt comfortable showing the QPL to physicians. A few did not due to lack of opportunity or concern about offending the physician.

I didn't show it to my doctor. I just told him that I had questions and kept going back to my phone… I don't want to let the doctor know that maybe he's not telling me everything he's supposed to

(02 ON Caucasian aged 45)

Most women said physicians were receptive to discussing QPL questions. Few women said the physician provided brief or dismissive answers to QPL questions, and only one said the physician refused to answer questions.

My doctor say that I have no time to take part in such activities because I'm a little bit busy, I have schedule, like appointments

(08 ON South Asian aged 31)

Women said the key enabler to QPL use was receiving it before a visit to plan which questions to ask. Other enablers noted by a few women included physician receptiveness, pleasing QPL design and print or electronic QPL options. Most women said they did not encounter barriers to QPL use, and commented favourably on QPL content and format.

I don't think there was anything that made it hard. For me, it was the first time that I had so many questions to ask, which felt good actually

(16 ON African aged 29)

Women identified three key benefits of the QPL: (1) preparing for physician visits by giving them insight into what to ask, increasing their confidence to initiate discussion and ask questions; (2) raising awareness of the link between HDP and CVD, increasing confidence and motivation to self‐manage their health; and (3) increasing knowledge of healthy lifestyle behaviours by prompting physicians to discuss how to prevent CVD.

I know that I need to manage [cholesterol] with lifestyle and now medication as well

(15 ON South Asian aged 41)

Most women said the QPL should be shared directly with women through pre‐ or peri‐natal clinics or classes rather than relying on physicians to share it with them. However, they recommended it also be shared with physicians to ensure that physicians were prepared for discussions.

Having the physician prepared to have the discussion and answer the questions before the patient coming…that would be extremely helpful

(01 AB Caucasian aged 37)

In summary, even though most women said their physician was receptive to the QPL, they recommended the QPL be shared directly with women in pre‐ or peri‐natal clinic settings rather than relying on physicians to share it with them, but also recommended it be shared with physicians so that physicians would be prepared for discussions with women. In this way, women could review the QPL before physician visits and prepare to discuss it with physicians. Women were in favour of a QPL that could be printed or saved to an electronic device to accommodate both preferences. These findings may be useful for planning a future trial.

## 4 Discussion

In this study involving 23 diverse women, most used the QPL about HDP before and during physician visits, prompting most physicians of differing specialties to discuss CVD risk reduction. As a result, most women rated their visit as highly person‐centred. Findings did not differ by demographic characteristics or recruitment method. Overall, it appears feasible to support post‐HDP patient–physician discussion of CVD prevention with a QPL.

Prior research revealed low knowledge of CVD risk among women with HDP [2, 3, 4, 5, 6, 7, 8, 9, 10, 11, 12, 13, 14, 15, 16, 17, 18, 19, 20, 21] and physicians [21, 25, 26] or demonstrated poor healthcare experiences of women with HDP [13, 19, 20, 22, 23, 24]. Despite considerable evidence of this problem, little research identified strategies to prevent CVD after HDP [27], particularly among high‐risk ethno‐cultural groups [5, 6, 7]. A review of 15 studies involving 1623 largely Caucasian women with HDP found they had little knowledge of CVD risk and a mere 4 studies evaluated educational interventions (print or online information, in‐person meeting) that assessed only participation rather than impact on knowledge or behaviour [48]. Thus, our research is unique because it pilot‐tested an intervention aimed at directly informing and empowering ethno‐culturally diverse women with HDP to seek care, and indirectly prompting physicians to discuss CVD prevention.

These findings raise implications for policy, practice and ongoing research. A few women said that physicians gave brief or dismissive answers and recommended physician education and sharing the QPL with them. This could be done in several ways. For example, a review of Canada's medical school curriculum revealed limited content on PCC or women's health; thus, the curriculum could be bolstered to increase awareness of this issue among future physicians [49, 50]. Given that a review of HDP‐relevant guidelines found that few included recommendations for managing CVD risk, guidelines could be strengthened by including the QPL as both a learning tool and discussion aid [51]. Participating women also recommended alerting women with HDP about CVD risk during pre‐ or peri‐natal interactions. Knowledge brokers including community health workers or midwives could share the QPL with women. In the context of a future trial, to support women who are dismissed by physicians, a nurse on the study team could reach out to discuss the QPL with those women, and the study team could disseminate an information package to those physicians including a version of the QPL with answers to each question.

Women scored moderately for self‐efficacy for self‐management and perceived self‐management. This suggests that women may benefit from additional self‐management support following QPL‐guided physician discussion. Considerable evidence shows that educational and digital hypertension and CVD self‐management interventions are effective and acceptable, even among populations prone to healthcare disparities [52, 53, 54]. Thus, a future trial could assess the impact on self‐management by including a link to self‐management resources in the QPL or having physicians or non‐physician knowledge brokers who review the QPL with women refer women to self‐management resources.

This study featured several strengths. We complied with established guidance for developing interventions [41] and employed rigorous research methods and reporting criteria for observational and qualitative research [37, 38, 39, 40]. The qualitative component yielded important information regarding QPL feasibility and corroborated quantitative results on how the QPL achieved PCC. Diverse women's responses were consistent, underscoring the relevance and reliability of the findings. A multidisciplinary team including ethno‐culturally diverse women informed study design and data collection and analysis. Participants were ethno‐culturally diverse, an important consideration due to their high risk of HDP‐related CVD [5, 6, 7]. Some limitations must be noted. The sample size was small but suitable for a pilot test conducted to inform a future trial involving a larger number of participants. Although women who used the QPL said they did not encounter any barriers, and most said that physicians were receptive to QPL‐informed discussions, recruitment was open so we did not assess the feasibility of recruitment by reporting the number of women at settlement agencies or prenatal classes who were eligible but chose not to participate. While CPCN physicians might be more amenable to discussing the QPL, most of the 13 women who saw their own non‐CPCN physicians reported satisfactory experiences. Still, non‐specialist physicians may be less receptive to the QPL, so as noted in the prior discussion, a future trial should provide support to women whose physician dismisses the QPL. We could not establish QPL impact but will do so in a future trial involving a before‐after design. The results may not be transferrable to women or physicians in other jurisdictions with differing healthcare systems.

## 5 Conclusion

This study demonstrated the feasibility and potential value of a QPL about HDP for empowering women and encouraging physicians to discuss CVD risk reduction, paving the way for a future trial.

## Author Contributions

**Smita Dhakal:** project administration, formal analysis, writing–original draft, investigation. **Bethany Rankin:** project administration, writing–review and editing. **Taryn Assaf:** conceptualisation, investigation, formal analysis, writing–review and editing. **Jane Baker:** investigation, writing–review and editing. **Laura Chisick:** investigation, writing–review and editing. **Tracey Colella:** conceptualisation, investigation, writing–review and editing, formal analysis. **Natalie Dayan:** investigation, writing–review and editing. **Maureen Dobbins:** conceptualisation, investigation, writing–review and editing, formal analysis. **Sherry Grace:** conceptualisation, investigation, formal analysis, writing–review and editing. **Serena Gundy:** investigation, writing–review and editing. **Sheila O'Keefe McCarthy:** conceptualisation, investigation, formal analysis, writing–review and editing. **Ziran Meng:** investigation, writing–review and editing. **Beth Murray‐Davis:** conceptualisation, investigation, formal analysis, writing–review and editing. **Sarah Neil‐Sztramko:** conceptualisation, investigation, formal analysis, writing–review and editing. **Kara Nerenberg:** conceptualisation, investigation, formal analysis, writing–review and editing. **Winnie Sia:** investigation, writing–review and editing. **Graeme Smith:** investigation, writing–review and editing. **Maria Timofeeva:** investigation, writing–review and editing. **Anna R Gagliardi:** conceptualisation, investigation, funding acquisition, writing–review and editing, writing–original draft, methodology, validation, formal analysis, project administration, data curation, supervision, resources.

## Ethics Statement

This study was approved by the University Health Network Research Ethics Board (lead site) and at all participating sites that required ethics approval.

## Consent

All participants provided written informed consent before taking part in the study.

## Conflicts of Interests

The authors declare no conflicts of interest.

## AI Use

No authors used generative AI and AI‐assisted technologies in preparing this manuscript.

## Supporting information

Supporting information.

Supporting information.

Supporting information.

Supporting information.

Supporting information.

Supporting information.

## Data Availability

All data generated or analysed during this study are included in this published article and its supplementary files.
