# Supplementary material for: Evaluation of a Question Prompt List About Cardiovascular Disease Risk and Prevention After Hypertensive Pregnancy: A Pilot Study
Source: Health Expect. 2024 Oct 30;27(6):e70085. doi: 10.1111/hex.70085 (PMC11522917; doi:10.1111/hex.70085)
Supplement: Supplementary file 1 — Supporting information. [file HEX-27-e70085-s005.docx]

Supplementary File 1. Question guide for qualitative interviews with participating women

KNOWLEDGE

| Question | Prompts |
| --- | --- |
| After visiting your doctor with the QPL, what do you know about how to prevent or manage heart disease? | Can you share what kind of things your doctor told you to do for heart health? e.g. healthy eating, physical activity |

MECHANISM

| Question | Prompts |
| --- | --- |
| How did you receive the QPL?  What did you do with the QPL when you received it? | Did you read it once or several times?  Did you think about the questions?  Did you write notes in the spaces provided? |
| Please describe how you showed the QPL to your doctor at the appointment | Did you feel comfortable doing that?  Please tell me more about that |
| How did your doctor react to the QPL? | How did your doctor use the QPL?  Did your doctor decide which questions to discuss or did they invite you to state the questions you wanted to discuss?  Were you able to ask QPL questions?  Did your doctor discuss answers to those questions with you?  Did your doctor give you the information you wanted?  Did they allow you to ask any additional questions? |

DETERMINANTS

| What made it hard to use the QPL? | I don’t know my doctor very well  I felt shy about showing the QPL to my doctor  My doctor was in a rush and did not look at the QPL  My doctor didn’t know the answers to the questions |
| --- | --- |
| What made it easy to use the QPL? | I received the QPL before my appointment so I had time to think about it  My doctor was showed interest in the QPL |

TIMING

| Do you think it is better for the QPL to be given to women before or appointments or should doctors give the QPL to women during appointments? | Please explain why |
| --- | --- |

IMPACT

| Question | Prompts |
| --- | --- |
| In what way did using the QPL help you? | How did using the QPL benefit you? Please elaborate  Know what to ask  Confidence to ask  Doctor paid attention to my questions  Doctor gave me useful answers and other information |
| In what other ways did the QPL help you or your doctor communicate about how to reduce heart disease risk? | Did the doctor give you brochures or pamphlets? On what topics?  Did the doctor suggest you speak to another type of doctor or other type of healthcare provider?  Did the doctor give you a list of web sites on the topic? |

IMPROVEMENT

| Question | Prompts |
| --- | --- |
| How could we improve the QPL? | Do you have any suggestions for the format?  Do you have any suggestions for the instructions or questions? |
| How could we help women like you to make use of the QPL? | What else would have helped you to use the QPL? |
