# Supplementary material for: Evaluation of a Question Prompt List About Cardiovascular Disease Risk and Prevention After Hypertensive Pregnancy: A Pilot Study
Source: Health Expect. 2024 Oct 30;27(6):e70085. doi: 10.1111/hex.70085 (PMC11522917; doi:10.1111/hex.70085)
Supplement: Supplementary file 2 — Supporting information. [file HEX-27-e70085-s006.docx]

Supplementary File 2. Consultation Care Measure scores for perceived person-centred care

| Person | Instrument items by domain scored on 5-point scale where 5 is highest agreement | | | | | | | | | | | | | | | | | | | | | Total score | Mean score | SD |
| --- | --- | --- | --- | --- | --- | --- | --- | --- | --- | --- | --- | --- | --- | --- | --- | --- | --- | --- | --- | --- | --- | --- | --- | --- |
|  | Relationship | | | Communication and partnership | | | | | | | | | | | Health promotion | | Positive clear approach to problem | | | Interest in life impact | |  |  |  |
|  | 1 | 2 | 3 | 4 | 5 | 6 | 7 | 8 | 9 | 10 | 11 | 12 | 13 | 14 | 15 | 16 | 17 | 18 | 19 | 20 | 21 |  |  |  |
| 1 | 5 | 5 | 5 | 5 | 5 | 5 | 5 | 5 | 5 | 5 | 5 | 5 | 3 | 5 | 5 | 5 | 5 | 5 | 5 | 5 | 5 | 103 | 4.9 | 0.4 |
| 2 | 3 | 4 | 1 | 4 | 4 | 5 | 5 | 2 | 3 | 4 | 4 | 3 | 3 | 4 | 1 | 2 | 2 | 2 | 3 | 2 | 2 | 63 | 3.0 | 1.2 |
| 3 | 5 | 4 | 5 | 5 | 5 | 5 | 4 | 5 | 5 | 5 | 5 | 5 | 5 | 5 | 5 | 5 | 4 | 4 | 4 | 4 | 3 | 97 | 4.6 | 0.6 |
| 4 | 5 | 4 | 4 | 4 | 5 | 5 | 5 | 4 | 4 | 4 | 5 | 4 | 3 | 3 | 3 | 4 | 5 | 5 | 5 | 4 | 3 | 88 | 4.2 | 0.7 |
| 5 | 4 | 3 | 4 | 4 | 5 | 5 | 5 | 4 | 4 | 5 | 4 | 4 | 3 | 4 | 5 | 4 | 4 | 5 | 5 | 5 | 4 | 90 | 4.3 | 0.6 |
| 6 | 4 | 2 | 4 | 4 | 4 | 4 | 4 | 4 | 4 | 4 | 4 | 4 | 3 | 4 | 3 | 3 | 4 | 3 | 3 | 2 | 2 | 73 | 3.5 | 0.7 |
| 7 | 5 | 5 | 5 | 5 | 5 | 5 | 5 | 5 | 5 | 5 | 5 | 4 | 4 | 5 | 5 | 5 | 5 | 5 | 5 | 5 | 5 | 103 | 4.9 | 0.3 |
| 8 | 3 | 4 | 4 | 3 | 4 | 4 | 4 | 4 | 4 | 4 | 3 | 4 | 3 | 3 | 4 | 4 | 3 | 3 | 3 | 4 | 4 | 76 | 3.6 | 0.5 |
| 9 | 4 | 5 | 4 | 4 | 4 | 3 | 4 | 4 | 5 | 4 | 3 | 5 | 5 | 4 | 4 | 5 | 5 | 4 | 4 | 3 | 4 | 87 | 4.1 | 0.7 |
| 10 | 5 | 4 | 5 | 4 | 4 | 4 | 5 | 4 | 3 | 3 | 4 | 2 | 2 | 4 | 4 | 4 | 4 | 3 | 3 | 4 | 4 | 79 | 3.8 | 0.8 |
| 11 | 4 | 4 | 3 | 5 | 4 | 5 | 5 | 5 | 5 | 4 | 3 | 5 | 3 | 3 | 5 | 4 | 4 | 5 | 4 | 5 | 4 | 89 | 4.2 | 0.8 |
| 12 | 4 | 4 | 3 | 4 | 3 | 4 | 5 | 4 | 5 | 4 | 5 | 4 | 2 | 2 | 4 | 4 | 4 | 3 | 2 | 3 | 3 | 76 | 3.6 | 0.9 |
| 13 | 4 | 4 | 4 | 4 | 4 | 4 | 4 | 4 | 4 | 4 | 4 | 4 | 4 | 4 | 4 | 4 | 4 | 4 | 4 | 3 | 4 | 83 | 4.0 | 0.2 |
| 14 | 2 | 2 | 4 | 2 | 4 | 4 | 2 | 5 | 3 | 4 | 4 | 4 | 3 | 3 | 4 | 5 | 4 | 4 | 5 | 2 | 3 | 73 | 3.5 | 1.0 |
| 15 | 5 | 5 | 5 | 5 | 5 | 5 | 5 | 5 | 5 | 5 | 5 | 5 | 3 | 5 | 5 | 5 | 5 | 5 | 5 | 5 | 5 | 103 | 4.9 | 0.4 |
| 16 | 3 | 3 | 4 | 3 | 4 | 4 | 5 | 4 | 4 | 3 | 4 | 4 | 4 | 4 | 5 | 5 | 4 | 4 | 4 | 2 | 3 | 80 | 3.8 | 0.7 |
| 17 | 5 | 4 | 4 | 5 | 5 | 5 | 5 | 5 | 5 | 5 | 5 | 5 | 5 | 4 | 5 | 5 | 5 | 5 | 3 | 4 | 4 | 98 | 4.7 | 0.6 |
| 18 | 4 | 4 | 3 | 4 | 5 | 4 | 5 | 3 | 4 | 4 | 4 | 5 | 3 | 4 | 5 | 4 | 5 | 5 | 5 | 5 | 4 | 89 | 4.2 | 0.7 |
| 19 | 5 | 5 | 5 | 5 | 4 | 4 | 4 | 4 | 4 | 4 | 4 | 4 | 4 | 4 | 4 | 4 | 5 | 4 | 4 | 2 | 4 | 87 | 4.1 | 0.7 |
| 20 | 4 | 5 | 4 | 3 | 4 | 4 | 4 | 5 | 4 | 4 | 4 | 5 | 3 | 4 | 5 | 4 | 4 | 4 | 5 | 4 | 4 | 74 | 4.1 | 0.6 |
| 21 | 5 | 5 | 5 | 5 | 5 | 5 | 5 | 4 | 5 | 5 | 5 | 5 | 5 | 5 | 5 | 5 | 4 | 5 | 5 | 5 | 5 | 103 | 4.9 | 0.3 |
| 22 | 1 | 1 | 4 | 4 | 2 | 2 | 1 | 4 | 3 | 2 | 2 | 3 | 3 | 4 | 4 | 4 | 3 | 2 | 3 | 3 | 2 | 57 | 2.7 | 1.1 |
| 23 | 5 | 5 | 5 | 4 | 5 | 5 | 5 | 5 | 5 | 5 | 5 | 5 | 5 | 5 | 5 | 5 | 5 | 5 | 5 | 5 | 5 | 104 | 5.0 | 0.2 |
| Item mean score | 4.1 | 4.0 | 4.1 | 4.1 | 4.3 | 4.3 | 4.4 | 4.3 | 4.3 | 4.2 | 4.2 | 4.3 | 3.5 | 4.0 | 4.3 | 4.3 | 4.2 | 4.1 | 4.1 | 3.7 | 3.7 | --- | --- | --- |

Individual mean score 4.1 (SD 0.2)

Overall item mean 4.1 (SD 0.6)
