# Supplementary material for: Evaluation of a Question Prompt List About Cardiovascular Disease Risk and Prevention After Hypertensive Pregnancy: A Pilot Study
Source: Health Expect. 2024 Oct 30;27(6):e70085. doi: 10.1111/hex.70085 (PMC11522917; doi:10.1111/hex.70085)
Supplement: Supplementary file 3 — Supporting information. [file HEX-27-e70085-s004.docx]

Supplementary File 3. Self-efficacy for Managing Chronic Disease scores

| Person | Instrument item score on 10-point scale where 10 is highest agreement | | | | | | Total Score | Mean score | SD |
| --- | --- | --- | --- | --- | --- | --- | --- | --- | --- |
|  | Item 1 | Item 2 | Item 3 | Item 4 | Item 5 | Item 6 |  |  |  |
| 1 | 8 | 8 | 7 | 8 | 8 | 8 | 47 | 7.8 | 0.4 |
| 2 | 5 | 5 | 8 | 8 | 7 | 7 | 40 | 6.7 | 1.4 |
| 3 | 5 | 4 | 4 | 6 | 5 | 6 | 30 | 5.0 | 0.9 |
| 4 | 5 | 8 | 7 | 8 | 7 | 7 | 42 | 7.0 | 1.1 |
| 5 | 10 | 8 | 8 | 8 | 6 | 5 | 45 | 7.5 | 1.8 |
| 6 | 7 | 7 | 4 | 6 | 4 | 4 | 32 | 5.3 | 1.5 |
| 7 | 8 | 9 | 9 | 9 | 10 | 9 | 54 | 9.0 | 0.6 |
| 8 | 5 | 6 | 7 | 7 | 8 | 8 | 41 | 6.8 | 1.2 |
| 9 | 5 | 6 | 6 | 7 | 9 | 9 | 42 | 7.0 | 1.7 |
| 10 | 9 | 9 | 7 | 8 | 10 | 9 | 52 | 8.7 | 1.0 |
| 11 | 3 | 7 | 6 | 8 | 9 | 9 | 42 | 7.0 | 2.3 |
| 12 | 6 | 6 | 8 | 9 | 7 | 8 | 44 | 7.3 | 1.2 |
| 13 | 8 | 7 | 7 | 7 | 8 | 8 | 45 | 7.5 | 0.5 |
| 14 | 7 | 6 | 9 | 9 | 8 | 8 | 47 | 7.9 | 1.2 |
| 15 | 10 | 10 | 10 | 10 | 9 | 8 | 57 | 9.5 | 0.8 |
| 16 | 7 | 5 | 5 | 8 | 8 | 8 | 41 | 6.8 | 1.5 |
| 17 | 5 | 7 | 2 | 2 | 8 | 9 | 33 | 5.5 | 3.0 |
| 18 | 8 | 6 | 6 | 7 | 9 | 8 | 44 | 7.3 | 1.2 |
| 19 | 6 | 5 | 5 | 6 | 10 | 7 | 39 | 6.5 | 1.9 |
| 20 | 7 | 8 | 8 | 9 | 8 | 8 | 48 | 8.0 | 0.6 |
| 21 | 8 | 8 | 9 | 7 | 9 | 9 | 50 | 8.3 | 0.8 |
| 22 | 8 | 8 | 7 | 10 | 10 | 7 | 50 | 8.3 | 1.4 |
| 23 | 9 | 9 | 8 | 9 | 9 | 9 | 53 | 8.8 | 0.4 |
| Item mean score | 6.9 | 7.0 | 6.8 | 7.7 | 8.1 | 7.7 | --- | --- | --- |

Individual mean score 7.4 (SD 0.6)

Overall item mean 7.4 (SD 0.5)

| Item | Item statement |
| --- | --- |
|  |  |
| 1 | How confident are you that you can keep the symptoms of HDP or heart disease from interfering with things you want to do? |
| 2 | How confident are you that you can keep any physical discomfort, pain and/or other symptoms caused by HDP or heart disease from interfering with the things you want to do? |
| 3 | How confident are you that you can keep the emotional distress (feelings like anxiety, depression or frustration) caused by HDP or heart disease from interfering with the things you want to do? |
| 4 | How confident are you that you can keep any other symptoms or health problems you have *other than HDP or heart disease* from interfering with the things you want to do? |
| 5 | How confident are you that you can do recommended things (exercise, healthy eating) to manage your health to reduce your need to see a doctor? |
| 6 | How confident are you that you can do things other than just taking medication to reduce how much HDP or heart disease affects your everyday life? |
