# Supplementary material for: Evaluation of a Question Prompt List About Cardiovascular Disease Risk and Prevention After Hypertensive Pregnancy: A Pilot Study
Source: Health Expect. 2024 Oct 30;27(6):e70085. doi: 10.1111/hex.70085 (PMC11522917; doi:10.1111/hex.70085)
Supplement: Supplementary file 4 — Supporting information. [file HEX-27-e70085-s001.docx]

Supplementary File 4. Perceived Medical Condition Self-Management Scale scores

| Person | Instrument item score on 5-point scale where 5 is highest agreement | | | | | | | | Total score | Mean score | SD |
| --- | --- | --- | --- | --- | --- | --- | --- | --- | --- | --- | --- |
|  | Item 1 | Item 2 | Item 3 | Item 4 | Item 5 | Item 6 | Item 7 | Item 8 |  |  |  |
| 1 | 4 | 2 | 4 | 3 | 5 | 2 | 2 | 4 | 26 | 3.3 | 1.2 |
| 2 | 2 | 2 | 3 | 4 | 3 | 3 | 1 | 3 | 21 | 2.6 | 0.9 |
| 3 | 4 | 3 | 3 | 4 | 4 | 3 | 3 | 4 | 28 | 3.5 | 0.5 |
| 4 | 3 | 3 | 2 | 5 | 2 | 3 | 3 | 3 | 24 | 3.0 | 0.9 |
| 5 | 2 | 4 | 3 | 4 | 3 | 2 | 2 | 3 | 23 | 2.9 | 0.8 |
| 6 | 4 | 4 | 2 | 3 | 2 | 4 | 4 | 2 | 25 | 3.1 | 1.0 |
| 7 | 2 | 2 | 4 | 4 | 4 | 2 | 2 | 4 | 24 | 3.0 | 1.0 |
| 8 | 3 | 3 | 3 | 3 | 4 | 3 | 2 | 3 | 24 | 3.0 | 0.5 |
| 9 | 4 | 2 | 4 | 4 | 5 | 3 | 3 | 4 | 29 | 3.6 | 0.9 |
| 10 | 4 | 3 | 4 | 5 | 4 | 2 | 3 | 4 | 29 | 3.6 | 0.9 |
| 11 | 2 | 2 | 4 | 4 | 4 | 2 | 2 | 4 | 24 | 3.0 | 1.1 |
| 12 | 2 | 2 | 3 | 3 | 2 | 2 | 3 | 3 | 20 | 2.5 | 0.5 |
| 13 | 2 | 2 | 4 | 4 | 4 | 2 | 2 | 4 | 24 | 3.0 | 1.1 |
| 14 | 4 | 2 | 4 | 4 | 4 | 2 | 2 | 4 | 26 | 3.3 | 1.0 |
| 15 | 2 | 1 | 5 | 4 | 5 | 1 | 2 | 5 | 25 | 3.1 | 1.8 |
| 16 | 2 | 3 | 4 | 4 | 4 | 2 | 2 | 3 | 24 | 3.0 | 0.9 |
| 17 | 3 | 4 | 5 | 3 | 4 | 3 | 2 | 4 | 28 | 3.5 | 0.9 |
| 18 | 3 | 2 | 4 | 4 | 4 | 2 | 3 | 3 | 25 | 3.1 | 0.8 |
| 19 | 3 | 4 | 4 | 3 | 4 | 4 | 4 | 2 | 28 | 3.5 | 0.8 |
| 20 | 4 | 3 | 3 | 4 | 3 | 3 | 2 | 4 | 26 | 3.3 | 0.7 |
| 21 | 2 | 2 | 2 | 5 | 4 | 2 | 2 | 4 | 23 | 2.9 | 1.2 |
| 22 | 2 | 4 | 4 | 4 | 4 | 2 | 2 | 4 | 26 | 3.3 | 1.0 |
| 23 | 2 | 2 | 4 | 4 | 4 | 2 | 2 | 4 | 24 | 3.0 | 1.0 |
| Item mean score | 2.8 | 2.7 | 3.6 | 3.9 | 3.7 | 2.4 | 2.4 | 3.6 | --- | --- | --- |

Individual mean score 3.1 (SD 0.3)

Overall item mean 3.1 (SD 0.6)

| Item | Item statement |
| --- | --- |
|  |  |
| 1 | It is difficult for me to find good ways to deal with problems that occur when I try to prevent or manage heart disease |
| 2 | I find that when I try to make changes to prevent or manage heart disease, they do not work well |
| 3 | I do things well to prevent or manage heart disease |
| 4 | I am able to do things to prevent or manage heart disease as well as most other people |
| 5 | I succeed in things I do to prevent or manage heart disease |
| 6 | Most of the time, the things I do to prevent or manage heart disease do not work out well |
| 7 | No matter how hard I try, things I do to prevent or manage heart disease do not turn out the way I would like |
| 8 | Most of the time, I am able to reach the goals I set to prevent or manage heart disease |
