# Supplementary material for: Evaluation of a Question Prompt List About Cardiovascular Disease Risk and Prevention After Hypertensive Pregnancy: A Pilot Study
Source: Health Expect. 2024 Oct 30;27(6):e70085. doi: 10.1111/hex.70085 (PMC11522917; doi:10.1111/hex.70085)
Supplement: Supplementary file 5 — Supporting information. [file HEX-27-e70085-s003.docx]

Supplementary File 5. Themes and quotes from interviews with participating women

MECHANISM

What did you do with the QPL?

| Theme | Recruited by physicians | Recruited in other ways |
| --- | --- | --- |
| Saved QPL and referred to it during interview | *Saved QPL on the phone*  I saved it in my phone under notes (02 ON Caucasian aged 45)  I read it a few times and then I had like the questions on my phone to ask her. I asked questions that I thought would be good questions, which was most of them because some of them I didn’t know like I didn’t even know that like the HDP could be related to heart disease so that was new for me, I didn’t know that. So I thought they were all good questions so [I asked all] (03 AB Caucasian aged 36)  *Printed QPL*  I printed it off and I brought it with me to my appointment and I looked at the questions and decided which questions I was going to go with (04 ON Caucasian aged 40)    I had a printed form. I had it with me during the [appointment] (05 ON Caucasian aged 35)  I presented it to my doctor’s appointment and I was able to have memorized the questions and I was reading them with the doctor. I had printed it (09 ON African aged 32)  I printed out and then I read all the questions. Then I said okay, those are good questions that I should discuss with her (13 QC African aged 42)  Yes I did print QPL (14 ON African aged 46) | *Saved QPL on the phone*  I bring the email [on my phone]. I review it and then have the review again (10 AB South Asian aged 46)  I did not print it. I made a note on my phone (16 ON African aged 29)  I left it in my phone. I showed him through with my phone (17 ON African aged 30)  *Printed QPL*  I printed it out and I wrote some things down myself. I printed out the sheet that had the answers on it and brought that to my doctor’s office as well and we kind of compared them (06 ON Caucasian aged 36)  Yah, I take a print-out (07 ON South Asian aged 40)  I read it over. I printed it and I put it into my purse so it was ready to go for the appointment (15 ON South Asian aged 41)  I just printed the document. I rather have the questions be printed rather than asking through my phone. Because of, you know. When you have your phone on, you can keep on getting calls. So I didn’t want any more interruptions (18 ON African aged 29)  Yes, I printed it to actually I have it at hand (19 ON African aged 30)  I printed out the sheet and I had already circled some of the questions I already knew the answer too (21 QC Caucasian aged 51) |
| Sent QPL to doctor before appointment | So I actually sent it to my doctor’s office and then I brought a copy in (23 ON Caucasian aged 30) | I also sent him a file [through email] for the for him to have a copy of what I was having (17 ON African aged 30)  When I received the document, I did actually go through it and yah, and I called my doctor since I had an appointment on Monday. Actually when we had the conversation, he did actually told me I should send the document to his email address. I emailed the document to the doctor. [He] see the questions and yah, right after I sent him, he told me that he was open (18 ON African aged 29)  I’m very friendly with my doctor so I booked an appointment over the phone, then I forwarded the QPL so my doctor, yah. So even before the appointment day, the doctor already had the QPL I had shared [emailed] (20 African aged 32) |
| Reviewed the questions before the appointment | I was like, oh my goodness this would be good to know. Oh I should ask this. Like I remember reading it and being like, oh I’m glad I have this (02 ON Caucasian aged 45)  I just reviewed it and then I thought which one, which questions would be good to ask my doctor, but I thought they were all good questions, so I asked her all the questions (03 AB Caucasian aged 36)  I picked the questions ahead of time for myself and I went with them (04 ON Caucasian aged 40)  When I received it from you, I like reviewed it all. So I had it with me like during the interview. I didn’t like take notes on it because I find that is it would have taken more time. Like I can’t concentrate as much, so I had it to kind of peek at and then discuss with him. I would have read it several times (05 ON Caucasian aged 35)  Before going to the doctor, so I prepare myself for sure. I read it and then when I went to the appointment (13 QC African aged 42)  I took my time to then go through them and then see the prompt Links that you had available and that really, really helped (14 ON African aged 46)  I reviewed the questions before my appointment and I filled in what I already knew. So things that are like how did I know I had hypertension or was like, you know I developed hypertension later in pregnancy and like I knew because of the blood pressure reading (22 ON Indigenous aged 26)  I reviewed it before my appointment a couple of times before my appointment actually… I ended up like typing it out on like a memo on my phone about what questions I wanted to ask instead of just kind of going to the doctor’s office and often times, sometimes you feel rushed because they’re maybe behind schedule. So I came prepared with like 5-questions that I wanted to ask and then I actually asked them (23 ON Caucasian aged 30) | So I, first of all I just go through the things. Like there are two, three pages. I have seen and I did little bit study on the Google and there are some links also. So I just searched few things (07 ON South Asian aged 40)  Yah I received it. I read those when I read those questions to like search it on the Google and just read those questions that what the, like what’s what is the study and is it matched with me or is it like match with my past experience, so that’s it yah (08 ON South Asian aged 31)  Before going to the doctor’s appointment I was able to read the questions and also like… be what anticipating to run from this session and know the topic… I was more prepared and more the, yah to learn (09 ON African aged 32)  I took a look and sort of like read up the questions and like prepared myself before I went to the doctor. I just sort of like wrote down what questions that I wanted to ask, what I needed more clarification on. And like what I needed to pay attention too, like the things that he was gonna say, like what I needed to like pay really close attention too (16 ON African aged 29)  I just read it through and then I investigated about all the issues that I had and I’ve gone through and I saw that most of things that were happening to me were related to it. And yah, that thought made me gain the courage to go and see the doctor (17 ON African aged 30)  Yes. Yah, I actually did go through the questions because I needed to like know what I’m; what we’re gonna discuss about; with the questions entailed. Yah, because I felt like some, some of the questions I actually didn’t know on how to go about it. So I felt like, yah it was actually something that I was having a conversation with. I just went through the question and I just ticked on some of the question that I’m gonna be asking (18 ON African aged 29)  I went through it first of all, and reviewed the questions that you had given me and tried to see if personally I do it through what questions whichever questions you are asking before I went to him. So first, I went through it before going to see the doctor (19 ON African aged 30)  Yes, I went through [QPL] (20 QC African aged 32)  I read [QPL] and I looked at a little bit about to understand better the link between heart, high blood pressure and heart disease (21 QC Caucasian aged 51) |

Please describe how you used the QPL at the appointment?

| Theme | Recruited by physicians | Recruited in other ways |
| --- | --- | --- |
| Did not show QPL to doctor | Oh I didn’t show it to my doctor. I just told him that I had questions and kept going back to my phone and asking him again or asking a different question… I just felt like a little shy because I don’t want to let the doctor know that maybe he’s not telling me everything he’s supposed to (02 ON Caucasian aged 45)  She physically did not see it. I just had the questions on my phone and then she just answered my questions (03 AB Caucasian aged 36)  I did not show him it but he saw it but I didn’t like give it to him. I kept it in front of me and asked the questions that way (04 ON Caucasian aged 40)  I just had it like with me. I didn’t actually show it to him or like have it, like out like right in front of me. So I had it like in my stroller so I could see it but it wasn’t like out on the table or chair beside me (05 ON Caucasian aged 35)    With my doctor, Actually, initially I wasn’t very comfortable because he’s always very busy and in a rush. So then thinking even though if he was going to agree to do it or not. [I showed it] when he looked at it and he said, oh yah, this is what it all it means. So he gave just a summary. So but initially I was like, oh my gosh, I don’t even know if he’s going to do it or not. I wasn’t very confident about it but he did take his time to read it actually, so that helped a bit (14 ON African aged 46)  I didn’t show it to him because he was very like he talked over me when I tried to questions and I just didn’t feel like there was a time where I could have discussed the tool even where like I just didn’t have that space (22 ON Indigenous aged 26) | I just sort of like asked questions referencing from my phone. I didn’t show him (16 ON African aged 29)  I didn’t show her the form but I just told her. I said, I have, [QPL] I’m part of a study. I have on the link between high blood pressure in pregnancy and heart disease and the goal is for me to learn better strategies or some strategies to try and reduce my risk of developing heart disease in my life. And so, I have some questions here. And I asked her you know if she was okay to answer them for me and she said, yah no problem… So I didn’t ask her all the questions but I circled the ones that I wanted to ask her and then I just read them from the sheet (21 QC Caucasian aged 51) |
| Felt comfortable to show QPL and ask questions | Yes absolutely [I felt comfortable]. I have a good relationship with my doctor. She’s a very good doctor, so I felt comfortable asking her. She is usually pretty inviting and willing to answer questions. So I didn’t feel any hesitation (03 AB Caucasian aged 36)  Yup [I was comfortable] he finished his spiel in our little discussion and then I was like, okay I have some questions and I pulled out the question prompt tool and looked at the questions and asked them in the order that I was interested and I went with questions; 3, 4, 5 & 6 and I had 7 & 8 too but he wasn’t too worried in the immediate moment about those two so I didn’t go really in depth on those ones (04 ON Caucasian aged 40)  I feel totally comfortable like bringing things like that or the tool into the appointment. I don’t feel like people would like judge me for that and I feel like sometimes people are like, oh that’s good, you like care and then you can understand the process and what’s going on and everything. So yah, I felt comfortable bringing it. I think it’s like useful, yah (05 ON Caucasian aged 35)  Yes, yes. [I was Comfortable]. At the end of the appointment, we did it. Once we finish, I ask her and I told her that I am participating in a study and then I have questions that I would like to ask her about what happened with the HDP and then she said, go ahead. And then, and I asked her how do I manage that when it happened at in February I didn’t have that before or why and what could cause that? How could I manage it? What could happen in the future? Stuff like that (13 QC African aged 42)  I showed him on the phone, on my cell phone the form that you sent (23 ON Caucasian aged 30) | Yah, I didn’t feel any hesitation to show it to them. I asked them about it when I made the appointment, so they knew that this was what I wanted to discuss so it wasn’t a surprise to them (06 ON Caucasian aged 36)  My doctor is very comfortable to discuss all these things. So I can freely speak to her about any issues…I just showed the [QPL] to my doctor and I told her that one of my friends from Saskatchewan, she just gave me these things because she also know that I was suffering from high BP issues in my pregnancy. So I just want to know more about that thing (07 ON South Asian aged 40)  For me I was comfortable because I’m coming from a place of where I want to learn and I was maybe I’ll say confident, that I would gain some knowledge after the session (09 ON African aged 32)  Oh yah, I was comfortable. That’s what I said, that I was okay. It’s okay. And then it was okay. That’s it (10 AB South Asian aged 46)  I went over all the questions with my healthcare provider during the appointment. I showed it to her briefly and then I read each question off and then we went over them together (15 ON South Asian aged 41).  Yes, I felt so much comfortable because the doctor really understands me and he’s so friendly, so I was able to share much with him and he was about to advise me whatever to do (17 ON African aged 30)  Yes, [I was comfortable] because we had that conversation before I came. So hearing how receptive and open he was it actually made me more comfortable to just stay open and ask… question I felt like I needed to know (18 ON African aged 29)  Yes, I was very comfortable. So, I think I have one the best doctors in Ontario. And my doctor doesn’t, like he always follow-up my health issue and for the time I have known him he doesn’t hesitate maybe… answering my questions. And so I’m very sure about this. And how I can report it, is he was free and any kind of questions that I popped up after reviewing your questions, he really give me the right answers and may be the answers I needed to hear (19 ON African aged 30)  Yes, I was [ comfortable]. We’re very friendly (20 QC African aged 32) |

How did your doctor react to it or use it at the appointment?

| Theme | Recruited by physicians | Recruited in other ways |
| --- | --- | --- |
| Physician provided brief or dismissive answers, or refused to answer questions | He didn’t really answer. I did ask it but I feel like I didn’t really get a clear answer because he’s saying, oh you’re already doing everything. Well, if I’m already doing everything, then why do I have it? And why did I develop it? He really couldn’t say…When I asked him like why does it risk my heart disease? He was just like, oh you’re probably fine. You’re okay than most of my patients. So I kind of felt, I guess I don’t need to worry about this? I think that’s probably why the next question I missed because it says, what are the signs and symptoms? But he kept telling me, oh you’re okay. And then when I asked him what is the risk of developing, he said, oh most people in your situation it’s like 33%, but yours is probably way lower than that. So, it kind of was like you have this problem, oh but you’re fine. I should have probably kept asking the question and repeating the question because sometimes they go off on a tangent and unless you repeat a question you don’t really know what the answer was. I guess in a way that’s my fail, I should have kept asking it but there was a lot of information being relayed and I probably needed to just go back and say, okay did we really answer this question (02 ON Caucasian aged 45)  I asked, what is my main risk in developing heart disease? That was more important to me. That’s when he had said, overall I’m a low-risk patient, but didn’t really tell me what that meant. So that was a bit confusing, like he didn’t tell me what different levels of risk, how I might go from low-risk to medium or high-risk, what the differences are between those groups. Most of like my family history has heart disease or hypertension. I shared that with him but he didn’t discuss how that might affect me and things that I can actually do about it. I found, when I asked questions it was he gave very short answers (22 ON Indigenous aged 26) | My doctor say that I have no time to take part in such activities and because I’m a little bit busy, I have schedule, like appointments. So I don’t, like I am not interested. That’s the main problem, that I couldn’t get answer from my doctor (08 ON South Asian aged 31) |
| Prompted physician to ask additional questions or provide more information | She was very informative. She would ask me questions as well. Like if I needed to know more information or you were comfortable with information or you needed more explaining. So, it was very good interaction (03 AB Caucasian aged 36)  She said, its really important if you feel something like mainly, like a chest pain and neck pain or heartburn or shortness of breath, its really important that I go may be see a doctor. I go to the emergency. I go to a clinic. Just to make sure that I am taking care of (13 QC African aged 42)  I showed it to him, he just glanced through it and then he quickly gave a summary and then he told me that because I have heart gestational diabetes, I’m at high-risk for cardio and then also because I had high blood pressure there’s a possibility of cardio issues. So that’s when he started giving the advice, maintaining a healthy weight and then eat right and exercise and sleep. And then he just said, and then we’ll keep monitoring it (14 ON African aged 46)  Doctor give me resources after about healthy eating. He gave me the title of a book that he had recommended I read and a pod-cast that he thought would be particularly interesting. He did ask me to participate in their gut health study and there was like resources on gut health with that (22 ON Indigenous aged 26)  Yes, there was like an information sheet… so he included like nutritional information. Like going over you know, making sure that what I put into my body, like what I eat is important and highlighted, like the different food groups that I should include in the ones that like highly processed foods that I should limit my intake and then making sure that I exercise and then do a variety of exercises including cardiovascular exercises but also strength training as well (23 ON Caucasian aged 30) | Yah they also looked at, it’s called a Framingham calculator which they had access too. I liked that it prompted my doctor to use that Framingham calculator because I’ve never of that before and I have seen my doctor about high blood pressure previously and no one had ever mentioned that. And based on previous blood work and previous blood pressure results I had in the office they were able to calculate a risk of heart disease for me over the next 10 years and they added that to the QPL, that result to the QPL (06 ON Caucasian aged 36)  He was asking me questions so I went through the questions. For example, he was asking me like which do I do to? What do I eat? Do I do any physical activities there (09 ON African aged 32)  Yes he give me a brochure. Yah, basically just about how one can be able to prevent heart diseases and some of the symptoms that I’m suppose to be looking out for. And also there is a contact number that was shared where we are able to like contact other doctors if he’s not on duty, yah (18 ON African aged 29)  Yah [He gave me pamphlet] That’s on for how you can reduce your heart diseases or how to manage your heart diseases (19 ON African aged 30)  She showed me some websites…I think it was the Canadian Heart and Stroke Foundation something, so that I could see more information about the recommended diets and also how to calculate my target heart rate (21 QC Caucasian aged 51) |
| Physician open to jointly review and discuss QPL questions | I was able to ask any questions I wanted too. He was very open to questions (02 ON Caucasian aged 45)  She invited me to ask whatever questions I wanted to do. Yah there was no limitations. I went through my list and then she would answer in the best way possible. And then she would ask me if I had any further questions or anything else regarding that question, like that she may have left unanswered and then we just kind of went through the list (03 AB Caucasian aged 36)  He was fine with it. I know he’s working with you on this, so he was really open to it and was interested. He didn’t ask me any questions about it. He just answered the questions that I asked him but [I asked] all the ones that I kind of picked. The rest [were] already touched on. I didn’t need to ask (04 ON Caucasian aged 40)  He kind of went over and like explained things to me initially and then he actually answered all of not all of them but like a good amount of the questions before I even needed to answer questions. So then there was just a couple questions that I had and he was able to answer them. I did ask but like he gave multiple like options. Like he asked me if I had questions like during like our appointment and then at the end too before we left he asked if there was any other questions (05 ON Caucasian aged 35)  She said, very well. She said those are good questions. She said, its good for you to know and I and she said I she encouraged every patient to take time to ask those questions and then she know that because I told her that it was a study and then she said, okay. This is very good questions that you should, every patient should ask because she saw more and more women with those kind of preeclampsia or HDP problem (13 QC African aged 42)  He reacted positively. He was like, oh yes, this thing. So like I said before, he knew what it was already which was nice and…I’m assuming that he was; he was ready for it… he kind of answered some of my questions before I even got a chance to kind of ask them. It’s almost like he just knew that what kind of questions I would have and then I was able to ask the remaining questions that I had (23 ON Caucasian aged 30)  I as | And yah they were really good about receiving it. They were happy to go through it with me yah. They went through each question with me and tried to decide certain things whether they pertained to me. For example, question 3; why does HDP increase risk of heart disease? And when we read through, they determined that I would fall into the number one category... They asked me if I had any other signs of heart disease (06 ON Caucasian aged 36)  First of all basically she, like there is a page which shows my HDP diagnosis. We talked about those things, then preventing heart disease. Then, because she is my family physician, she knows that I have a family background history for heart disease even my mom, she also went through the brain stroke. So she know the history of my family, so she was interested to remove my curiosity so that I can be more comfortable. I can be more sure that what I need to do to prevent such things for the in future with me or my family (07 ON South Asian aged 40)  I presented the document and I told, like I wanted to learn more about high HPD. The doctor was or maybe amazed first of all because maybe they don’t expect patients to do such a thing. But he was happy because I’m someone maybe wanted to learn more so that I can protect my health. like I had carried a document and also how I wanted to learn more but I could see from his facial expressions, like he was happy because first of all, I’m going, like I was, it’s like something that I’m going to learn and for, it felt like he was even interested in sharing more because it came from a point to where like it’s more individual right, yah. Okay like he read through the document and then he was asking me about my condition and then I was explaining like I’m a woman who has high blood pressure and then I felt I was more willing to share, like throughout the appointment. Yes, he was reading through the questions and then asking me about any [questions] which I have and then like inviting me back (09 ON African aged 32)  She asked me that if anything first she, anything I need additional help or any [questions]; do I need and then also she wanted to know that specific, what part I need [answers]. I need help right now. And then I say that, I need those you know the thing you send me. And then it’s all actually I know the answer of those already. Me also know and then doctor said gave me like additional information overall (10 AB South Asian aged 46)  She came in and I showed her the questions and I just; she looked at it briefly but then I posed each question one at a time. As she gave her answers to the questions, I noted them down on the QPL and we discussed any further if we felt like we needed to. I asked every single one of them. We discussed answers to every single question (15 ON South Asian aged 41)  I think at first, he was like, he felt like I was well prepared and like I was generally concerned about my health. And he even with like a joke about how I have so many questions but he was very forthcoming and he was very appreciative of the fact that I had done prior research and not and not like just gone without any preparation (16 ON African aged 29)  First of all, he was so pleased about it because he was like… you are taking of care it and you know you are trying to know more about your body and also to take concern about your life. And he was so much happy and just I’m going to an extra extent of investigating more about the HDP (17 ON African aged 30)  When I went to the doctor’s office we just simply went through the questions. He just told me which questions are… like would you want to know more on? So yah, that is what; that is actually went on that day, yah (18 ON African aged 29)  So it was in a way that oh, where did you get it and what are you seeking answers for? So I just put it how he ask for me. So actually he asked me to re-review each question or you just want to pick point the question. So let me say this, there was a patient behind me and the patient interrupted the doctor and doctor was like why don’t I treat that one and then you come after and we go through the questions each. So he treated another patient and then I went back and going back we used every questions (19 ON African aged 30)  The doctor knew everything; okay, whatever made me book the appointment. So, he heard all the answers of other questions I had that time. The doctor was very impressed actually I really wanted to know something and to just answer; a prevention before maybe I get the disease or something. All the questions I had about whatever I can do at home, whatever I can eat, whatever I should avoid the doctor shared everything (20 QC African aged 32)  [The doctor] was very opened to answering all the questions and if I had follow-up questions, if I didn’t understand, she went into detail and she showed me stuff on the internet and she was very helpful (21 QC Caucasian aged 51) |

DETERMINANTS OF USE/IMPACT

What made it easy to use the QPL?

| Theme | Recruited by physicians | Recruited in other ways |
| --- | --- | --- |
| Receiving QPL in advance of appointment | One of the things that made it easy is that it was provided to me ahead of time. So I was able to read the questions and see which ones were applicable to me and which ones I didn’t need to spend as much time on (01 AB Caucasian aged 37)  I just used that [QPL] as my method of question asking. If I didn’t have that, I would have thought of no questions. I would have just gone in there and just listened (02 ON Caucasian aged 45)  That you gave it to me, like thankfully I even had it. I wouldn’t have thought to ask any of these questions. So you sent it to me. I need to bring this to my appointment and use this to ask questions with the doctor (02 ON Caucasian aged 45)  I had the questions on hand because I might have not thought of to ask those questions to my doctor. So, I thought it was a good tool because it gave me awareness of what to ask… it made me interested in it because that obviously affects my health and knowledge is power so it’s good to know that stuff (03 AB Caucasian aged 36)  It was nice to have those questions there because sometimes when you get in there you get overloaded with information and then it lets you sort of slow things down. Having it ahead of time helped because then I could write any other questions I had (04 ON Caucasian aged 40)  Having it before was really helpful because… you can look at the resources to understand hypertension, so you kind of understand a little bit about the questions that you would want to ask and discuss at the appointment instead of just going into the appointment completely blind (05 ON Caucasian aged 35)  Just because I was at ease and I read the questions before. It help me guide the conversation with the doctor because if I didn’t have all those questions, it could have been very hard for me to go deeply into the subject and ask the right questions. I think it’s a very important, very well relevant (13 QC African aged 42)  To read in advance and like type out my own answers (22 ON Indigenous aged 26)  Having it before definitely helped… I wouldn’t haven’t had the chance to come up with the questions. So definitely getting it beforehand was excellent (23 ON Caucasian aged 30) | Then I got it, I was not aware of all those things. So now, that made me to curious to book an appointment with my doctor because I thought it was helpful for me (20 QC African aged 32)  I guess the fact that I got it ahead of time, so I was able to read the questions and do a little bit of research myself so I understood some of the basic ideas (21 QC Caucasian aged 51) |
| Physician was receptive to using QPL | She was very receptive. The doctor was prepared to have a discussion and reviewed the questions that were applicable to me (01 AB Caucasian aged 37)  [The doctor] was very invested in it. She was very interested in my questions and wanted to make sure that I had an understanding. And she would always ask me if the information was clear, or she wanted you know describe it in another way. Like making sure that I understand and making sure that she answered all my questions (03 AB Caucasian aged 36)  She said, those are questions every patient should ask, very good questions and then I encourage you to do anything that is suppose to prevent heart disease, and then we talk about it. It was easy, it was easy. She took all the time with me and then we go over it. I didn’t have any, any problem using it, not at all (13 QC African aged 42) | She was interested to discuss all these things with me. So, she took almost 15 to 20 minutes. She’s also interested to give answers to my queries and she is not in rush. So, she was listening to me. She was giving attention to me (07 ON South Asian aged 40)  The doctor allowed it. I’m saying it was easy for me to use it with my doctor (09 ON African aged 32)  Just with the way he was answering, he wasn’t just giving like one statement answers, he was diving into the details and making sure that I understood, and even when I was done, he was like, are you sure you don’t have anymore questions (16 ON African aged 29)  The doctor was able to have the interest to even follow it and get in touch with it (17 ON African aged 30)  He was actually quite receptive. That is something that actually made me feel more comfortable to ask the questions (18 ON African aged 29)  The doctor had interest in the questions so that made it easy for me and for him (19 ON African aged 30) |
| Print and electronic format available to suit different preferences | Well I mean I just downloaded it onto my phone so that made it really easy for me to just bring in with me (02 ON Caucasian aged 45)  I liked using it on the phone and I thought that was the best format for me anyways. Having it be compatible with a cell phone is important… if it was in a format that I couldn’t open on my phone I probably wouldn’t have used it. Like if I had to print it out and that was the only option then I would have been out of luck because I forgot it. But I was able to get it on my phone (23 ON Caucasian aged 30) | I think the easiest thing was having it printed. Like if, I had it on my phone I think it would have been harder and kind of, I don’t know more of a distraction like pulling out your phone during an appointment just doesn’t feel right. But I think having it printed and having how it's kind of laid out like in the 4 sections with two questions I think having it just simplified within those questions makes it the easiest (05 ON Caucasian aged 35) |
| QPL design features (not too many questions, questions clear, space for answers) |  | The questions were laid out nicely on the sheet and there was decent space to fill in things, they were clear. The questions were clear. It just made it easier to have the answer sheet to have a guide to go off of (06 ON Caucasian aged 36)  I would say it was brief and it was very well detailed. The questions were not a lot and the questions were straight (09 ON African aged 32)  I felt like the questions are direct, to the point, and I felt like they were the right questions to ask (18 ON African aged 29)  It was readable. There was nothing to strain about (19 ON African aged 30)  There was spaces for me to write stuff, that was helpful (21 QC Caucasian aged 51) |

What made it hard to use the QPL?

| Theme | Recruited by physicians | Recruited in other ways |
| --- | --- | --- |
| Did not encounter any barriers | I actually had no difficulty using the QPL. It was very straightforward and I liked that the sections were kind of broken down so I could choose which ones to really discuss (01 AB Caucasian aged 37)  I wouldn’t say there was anything hard about it at all. I thought it was pretty informative. So I thought it was a good tool, so didn’t find it difficult (03 AB Caucasian aged 36)  I didn’t have any problems. For me, it wasn’t hard to use it (13 QC African aged 42) | For me, I would say it was not hard to use it. It was brief and it was very well detailed (09 ON African aged 32)  I am feel right now that I don’t feel it hard (10 AB South Asian aged 46 )  Nothing made it hard. It was very easy (15 ON South Asian aged 41)  I don’t think there was anything that made it hard. For me, it was the first time that I had so many questions to ask which, which felt good actually (16 ON African aged 29)  I actually don’t think there’s something hard about it because I felt like the questions were quite clear right to the point. So I don’t think I did have any challenge (18 ON African aged 29)  There wasn’t any hard thing to use it (19 ON African aged 30)  It was not too hard (20 QC African aged 32)  No, it wasn’t hard for me to use (21 QC Caucasian aged 51) |
| Doctor being in rush and not interested to answer questions | It’s just that he’s always in a rush. I always feel like when I go to the clinic he’s in a rush If I go, something is bugging me, he just goes, oh its nothing. To me it shows like he’s shutting you down instead of allowing you to actually express what you’re worries are. For most of my issues, I find he brushes things aside very easily (14 ON African aged 46)  The doctor, he didn’t let me talk very much in the appointment. And he was on a roll about eating fiber and this gut health thing. So by the end of the appointment, he was basically running out of time. He didn’t seem like he wanted to talk to me very much (22 ON Indigenous aged 26) | The questions in the QPL is really simple. So if she wanted to answer it… those questions take 10 to 15 minutes, but she said that she’s in rush, so she’s not interested (08 ON South Asian aged 31) |

IMPACT

Knowledge about healthy lifestyle behaviours

| Theme | Recruited by physicians | Recruited in other ways |
| --- | --- | --- |
| Engage in physical activity | Things that I’ve discussed with my physician at the appointment in terms of how to prevent heart disease in my case would be first of all, to engage in more physical activity, about 30 minutes, at least 30-minutes a day (01 AB Caucasian aged 37)  You want to get at least an hour and 30 minutes a week of some good exercise, something that would raise the heart rate a bit (03 AB Caucasian aged 36)  He said for the physical activity it didn’t need to be anything like specific. Like I have two kids, he knows that it’s hard to be physically active to kind of put time to like to go to the gym but it just has to be simple things, so like walking down the road. He gave the example of I live in the country so there’s telephone poles. So, like walk fast or get to running one telephone pole and then walking between the next two to kind of get your heart rate up because getting the heart rate up is very good. So simple activity, just walking is very important (05 ON Caucasian aged 35)  Exercising at least 30-minutes a day (13 QC African aged 42)  The exercise component which is actually a lot less than the nutritional component but still you know making sure that I exercise regularly is important in preventing heart disease (23 ON Caucasian aged 30) | I know that I need to keep physically active (06 ON Caucasian aged 36)  We have to do the exercise and it can be walking or jogging or you can go the gym or it can be a yoga, so these are the few things we can do to prevent the heart disease (07 ON south Asian aged 40)  She recommend me to do the exercise... Yah she told me that join any yoga class or any like walk or any exercise, like light exercise start with that, that’s it (08 ON South Asian aged 31)  Being physically active is also a key thing. My doctor encouraged me to engage in physical activities like even when I am at home, like I can try to go to the gym. I can also try to do like morning Also like, being like exercising to increase my body fitness level or increase may be system and make my body more efficient (09 ON African aged 31)  To maintain a healthy lifestyle and as well [be] active… I have to include activity in my life like which I do actually because in the winter time I just, I aware myself, like everything, just I’m thinking that okay about the heart, because I’m thinking that okay I should do you know, base this. But later I realize no, I can do my every; like I can do my work. I can have my normal lifestyle. Just I need to follow several things. Like I choose now-a-days, my work is like walking more in walking (10 AB South Asian aged 46)  I know that I need to manage it with lifestyle [like] exercise (15 ON South Asian aged 41)  Like exercising. Just like try and stay active, like maybe take more walks or like have like a; like what he was recommending is like get a smart watch and then try and then like track my steps and I have a target and just try and make lifestyle changes that will slowly be incorporated and collect my everyday routine (16 ON African aged 29)  Okay most of things is staying active. You do some physical exercise ( 17 ON African aged 30)  And also being active like exercising for me because I’m a stay-at-home mom so I don’t get like travel more options. So again, encouragement that I should; I’m supposed to be like walking at least 20-minutes in a day or 20 to 20-minutes in a day, yah (18 ON African aged 29)  I should be able to work out in the morning and preferably in the evening but instead a lot of during the morning time (19 ON African aged 30)  To be physically active. The doctor suggested that I do some walking like daily. I should avoid being idle and do just any, any house chore that will make me physically active (20 QC African aged 32)  So we talked about exercise and how to achieve my target heart rate and what that means and how to calculate it and what the required, how often to do that…[my doctor] told me that ideally I do 30 to 60-minutes, 3 to 5 times a week and with aiming to get my target heart rate in while I’m doing that (21 QC Caucasian aged 51) |
| Eat a healthy diet | Try to adopt a more healthier diet like a Dash diet or a Mediterranean diet because my husband is Asian and he cooks a lot of food with sodium, so trying to reduce my sodium intake (01 AB Caucasian aged 37)  The doctor gave me a few foods that I could put in my diet that I was already eating [like] sauerkraut, kefir, kombucha but not with sugar, pretty much gut-friendly foods. He mentioned like not a lot of red meat. He said that I should avoid baked goods… avocado oil wasn’t that great and just stick to all olive oil (02 ON Caucasian aged 45)  I would say the best prevention would be diet, eating more healthier. Basically they said to maintain a healthy weight, eat a good, have a good diet not like you know not dieting but like you know stay away from sugary items or you want to eat complex carbohydrates, things like that (03 AB Caucasian aged 36)  [The doctor advised] Just the diet to look at my diet (04 ON Caucasian aged 40)  For diet, he discussed just keeping it simple like cutting out processed foods like obviously, like treats and stuff but also eliminating things like processed like breads and rice and things like that and increasing more like vegetables and low processed foods is going to be the most impactful for me (05 ON Caucasian aged 35)  Manage my diet. Eat more vegetable, less greasy food (13 QC African aged 42)  Eat a healthy diet (22 ON Indigenous aged 26)  I now know is nutrition and what exactly that means in like including like a variety of different things in the diet including like fermented things like kabocha or like kimchi, making sure everything is like well-rounded. You know limiting sugars and those high processed food is really important (23 ON Caucasian aged 30) | I need to maintain healthy diet. They just said that I need to do that (06 ON Caucasian aged 36)  I discussed about heart disease with my doctor, she told me that we can prevent it by maintaining a good life on the daily basis. Like we have to be careful about the food. She told me that as I am vegetarian. So she told me that you can add a protein to your breakfast or lunch and dinner should be light. She suggested to take fruits and green vegetables and she also suggested me to have daily products but not too much and she said that we can; I can also have a fresh juices made at home. She told me to avoid junk food from the life because my father has a history of heart disease. So she told me that I have to be more careful (07 ON South Asian aged 40)  She recommend me to eat health food or balanced diet (08 ON South Asian aged 31)  Okay after the visit I’m able, like to know like eating well, like a I’ve been a balanced-diet and be key to preventing HDP or so and getting in harm for the healthy eating, I felt I was encouraged to have foods which are of low-fat and also high fibre content. And I would eat plenty of fruits and vegetables (09 ON African aged 31)  I remove sugar and you know fat things. I remove all and then I’m getting now those, yes sometime I just you know like if I craving a cheat that is the different, that is more; that’s really different. But most of the time my goal is to you know go with the healthy one to really it’s helping me (10 AB South Asian aged 46)  leading a healthy lifestyle, diet, high diet, rich in vegetables, low cholesterol (15 ON South Asian aged 41)  Right now, I know like my best course of action is to stay active by regularly exercising and like just having healthy habits, also eating well and avoiding a lot of processed food and stuff like that. Things that’s like high in cholesterol and stuff like that (16 ON African aged 29)  Yes most of the time you’re suppose to eat food that do not have a lot of fat and ensure that you eat fruit and vegetables so much so that you can balance of the diet and ensure that you also eat more of the plant protein rather than the animal protein( 17 ON African aged 30)  Yes, he told me about healthy eating. That I should be doing a lot of veggies and I should reduce meat forms (19 ON African aged 30)  We talked about reducing salt in my diet and just in general, the Mediterranean diet and another one whose name I can’t remember are Dash diet are ways to adapt ones diet to improve heart health (21 QC Caucasian aged 51) |
| Get better sleep | Try to have good sleep, try to have at least 6, 7 hours of sleep (13 QC African aged 42) | I need to sleep more. I need to get better sleep (06 ON Caucasian aged 36)  Good amount of sleep because sleep really affect my high blood pressure if I don’t sleep well, I really feel really, really, really bad even whatever I’m doing, no matter aside from my you know a good sleep that one (10 AB South Asian aged 46)  Healthy sleep routine and oh sorry, healthy sleep and routine care (15 ON South Asian aged 41)  Get a lot of sleep (20 QC African aged 32) |
| Reduce stress |  | I don’t need to have stress or anxieties. I have to be keep and calm, I have to make myself happier (07 ON South Asian aged 40)  She recommend me that my stress level also decrease (08 ON South Asian aged 31)  So he told me that I’m suppose to like find ways to manage stress. If I’m stressed out I can keep a journal and write my thoughts. I can talk to someone. Yah, other times I can also do yoga or meditation (18 ON African aged 29)  I should reduce the stress that normally affects me. Like managing of my stress, he’d tell me that over stressing yourself actually can bring high blood pressure (19 ON African aged 30)  Avoid anything that gonna stress you (20 QC African aged 32) |
| Control cholesterol | *Control cholesterol with medication*  My cholesterol has become slightly elevated so there is a potential if it does not change with the changes in the diet to also start taking certain medication to help me lower the cholesterol level (01 AB Caucasian aged 37)  There is also preventative there’s also medication that can help too (03 AB Caucasian aged 36)  *Control cholesterol with diet and exercise*  To lower my cholesterol with the diet and exercise. I have cholesterol level that could be medicated, but he thinks it can be controlled with diet and exercise, and he was gonna send me a letter explaining a treatment plan (04 ON Caucasian aged 40)  Well I think for me the like most concerning thing was my cholesterol. So, it was our conversations were mainly focused about that. So for me personally it was about increasing my physical activity and eating a healthier low processed diet to manage my weight and to decrease my cholesterol (05 ON Caucasian aged 35) | *Control cholesterol with medication*  I know that I need to manage [cholesterol] with lifestyle and now medication as well. Adhering and complying to medications (15 ON South Asian aged 41)  *Control cholesterol with diet and exercise*  He told me about healthy eating. So the workout. I should reduce my cholesterol (19 ON African aged 30) |
| Monitor blood pressure at home | Continue to monitor my blood pressure (22 ON Indigenous aged 26) | They told me to use my at home blood pressure monitor more and keep recordings of my results. They told me when it gets to a certain point consistently to call them and discuss further treatment (06 ON Caucasian aged 36)  Yes, there was also conversation on how often you’re suppose to like monitor your heart health because I feel like, so that has all the information on it. Actually quite it’s so and no one actually keeps tabs on that. They don’t know like how often you’re suppose to like be doing that. [Doctor suggested] keeping tabs with my blood pressure or yah, I should always watch [if] my blood pressure is up (18 ON African aged 29) |
| Maintain body weight | Control the weight (13 QC African aged 42) | The most important thing, it’s if I discussed that my weight is like really, like my weight is gaining, so and after pregnancy its like frequently gained. So the main thing she recommend me is changing my lifestyle (08 ON South Asian aged 31)  Ensuring and able to maintain healthy weight for my body and also cutting on consumption of alcohol and yah (09 ON African aged 32) |
| No smoking | No smoking. Don’t smoke (13 QC African aged 42) | I know that I need to manage it with regards to you know smoking and drinking and all of that. I’m aware of what I need to do in order to mange it (15 ON South Asian aged 41)  You avoid smoking (20 QC African aged 32) |
| Avoid alcohol |  | You should try to avoid the alcohol (20 QC African aged 32) |

Helped to prepare for physician visit

| Theme | Recruited by physicians | Recruited in other ways |
| --- | --- | --- |
| Helped to know what questions to ask | I found it extremely helpful. In general, it kind of prepared me to have that discussion with my physician. It gave me an idea you know what would be the first questions to ask her and which questions applied to me more and which ones didn’t about how to potentially prevent hypertension in the future and the route to prevent heart disease… So we were able to discuss you know potential diet plans, exercise and medication choices (01 AB Caucasian aged 37)  [QPL] definitely made it a little bit easier going in… seeing it on this paper. It’s like okay, well I’m kind of ready to know what that means when they say it. It means preeclampsia or and gestational hypertension (02 ON Caucasian aged 45)  So having like a bit of an understanding before you go to the appointment. Having an idea of what will be discussed at the appointment and then if things are not discussed you can be like, oh but I have this question, so you can kind of bring the conversation back to like, oh why did I develop this? I think like that was one of the questions that wasn’t answered initially when he kind of just did the overview so we could go back to that and be like, oh he thinks I developed this because of this. So it was nice to have so I could go back to that question and be like, oh but why? So I really liked having it because I think the majority he was able to answer without me like prompting with the questions but that was one that wasn’t so I was able to go back. And that might not have been something that I would have asked if I didn’t have it with me (05 ON Caucasian aged 35)  You already have those questions and the way that that the questions are formed, so you are able to ask the right questions and get the right answers (13 QC African aged 42)  It helped me frame like which questions I wanted to ask and what I thought was important. So like I had; I’ve had like preeclampsia in both of my pregnancies and in the first one, like I didn’t get any follow-up but this one I did. So I was actually able to read some of the stuff on like why it happens and then being able to actually ask that question of like why and how and like when does (23 ON Caucasian aged 30) | Even I have something in my mind but sometime I, may be I was unable to ask to my doctor. So through the QPL I am more concerned about the questions that yah, this is a question that was in my mind but how to ask from the doctor in medical terminology, so this help me. The QPL helped me to find out what I really need to ask to my doctor, yah (07 ON South Asian aged 40)  Yes, I would say because of [QPL], maybe like have the questions, like we had something to discuss, so like I will; we knew like what to talk about when you finish this question, the next (09 ON African aged 32)  Yah it helped. It helped during, yah. So I had the idea what to ask. Because I know that what we; what things are going to be asked, right (10 AB South Asian aged 46)  It helped me know the right questions to ask and which now translated to having the right information given out to me because I had the right questions and I felt like I was even informed even before I went and was being more informed after (16 ON African aged 29)  So I really went there with the knowledge of knowing what question or what to [ask] what am I suppose to do or what I’m, what question am I suppose to ask my doctor before I even go home and try to control or manage (17 ON African aged 30)  I feel like having the document actually made it easier for me to like just ask the right questions. The questions that I feel like I just needed answer on… in regards to my daily to daily schedule (18 ON African aged 29)  It’s helped me. I didn’t have to think very hard about what questions to ask, like it was all written there so it made it simple for me to ask the relevant questions and also the websites on the bottom provided useful links for me to do a bit of research ahead of time (21 QC Caucasian aged 51) |
| Helped women initiate a conversation | It actually helped shape and guide the conversation in terms of, first, we talked about how to identify heart disease in the future, and then, what are some of the techniques to prevent it. So it helps keep a conversation going and flowing instead of jumping around from one thing to another (01 AB Caucasian aged 37)  Okay, well I think the best improvement is that it brought it up into communication and it got the conversation started and then in that way we talked about prevention and ways to; for prevention. So I thought that was very effective. And I think without the QPL we wouldn’t even have the conversation started or any preventative actions discussed. So I thought it was helpful in that way to get the conversation started and to for her to pass her knowledge onto me (03 AB Caucasian aged 36) | It helped talk about some of the signs of heart disease which some of them I didn’t know like sweating. That prompted a conversation with my doctor about that. It helped us to go through some of the risk factors which I didn’t really fall into any of the ones included on the question prompt tool (06 ON Caucasian aged 36)  Like the questions was really educative and… maybe it helped the conversation flow with the doctor. Like we knew what you are talking about and also during the session we were able to have a good interactive conversation on the topic (09 ON African aged 32) |
| Increased confidence to self-advocate | I felt more confident to ask questions. Before I would have just sat there and listened to what he had to say. And I mean I did ask him some questions, like I tried. I reminded him like you saw me twice while I was pregnant and my legs were swollen all the way up to my sides and you told me that that was normal and nobody diagnosed me with hypertension disorder at that time and everybody just kept saying, oh that’s normal in pregnancy (02 ON Caucasian aged 45)  I think it allowed me to it said ask the questions that I wanted to know and make sure I got all of the information I needed which gave me more confidence and knowledge in how to treat it going forward (04 ON Caucasian aged 40)  And it also like makes you feel like actually involved in your care. Like oh, I have power to actually change and like I can actually be the one that; I’m the one that’s really in charge of my care because I’m the one that has to do the work to change this and like, I don’t know. I think it can be motivating for some people to feel more involved in their care then just going in and sitting and listening to a doctor (05 ON Caucasian aged 35)  Even after the whole pregnancy thing was over he did monitor the blood pressure once in a while when I go in for a visit. But then once the numbers were okay, everything was like, it was in the past. But then having as discussed it, it’s not like an open thing that makes it easier for you to know that okay, he knows that you are at risk for this. So anytime you go, when he’ll be monitoring you more to make sure that you are not developing any of the symptoms. For now, that’s what I think. But if it actually happens, that’s a whole different thing. But I do think that QPL help open both of us onto one page to say that okay, this is … problem for you and we’ll keep monitoring… now it’s like two people looking at the same thing instead of just one person thinking about it (14 ON African aged 46)  It helped me like to prepare for my appointment and it helped me, like to not have as much anxiety about the appointment. I had a lot of like anxiety leading up to the appointment and not knowing what we were going to discuss or you know how, you know it gave me a foundation to think about my own situation and questions that I should ask and it did ease some anxiety about that appointment (22 ON Indigenous aged 26)  I think its because at least for me anyways, like I felt like I said before, I walked in knowing what ones like what questions were important to me which made me feel like I was just kind of in control at least of how and like what I wanted to ask instead of just responding to the doctor. Often times you know that doctor patient relationship is kind of like the doctor’s in control of it. Like they’d give you the information but at least like you; like that form gave me the question, like an idea of what questions I wanted to ask which made me walk into it confident but also walk out of the doctor’s office saying, I was able to ask the questions that I wanted to ask and not forget them and have the information that I felt was necessary to better my health (23 ON Caucasian aged 30) | I’m more confident yah (10 AB South Asian aged 46)  The questions was straight forward and the questions felt like they were giving me power and they were like making me very informed even prior to like my appointment (16 ON African aged 29)  I was able to ask… chat with the doctor and I was able to be guided well and again, I had the psychological relief from using the QPL because I had actually read through it and so it was much easier for me to see a doctor (17 ON African aged 30)  I was able to know about my HDP… advice they… a positive note because … ours been very negative about it and you were talking about high blood pressure in pregnancy actually… only me, but during these research I found out there are so many things and so many people struggling with it and I wasn’t the only one. So, I think that really helped me out. The courage and the confidence of asking (19 ON African aged 30) |
| Increased my awareness and knowledge about link between HDP and heart disease | I just felt like I knew more. Like I felt like I had knowledge of what it even related too. Like I don’t think I kind of I didn’t really realize that that was related to heart disease. I think it just helped me to realize that like I had issues. I probably would have just overlooked a lot of things and so it made me focus on some of the stuff that I needed to focus on before that appointment (02 ON Caucasian aged 45)  I thought it helped a lot because like I said, I didn’t know that the high blood pressure would have you know might cause heart disease. I didn’t, I wasn’t aware of that at all until I did this study. So, it did improve in the way that I have awareness about it and then I have prevention about it and to be aware that there is testing and medications that can be also applied to the situation. So I thought it was very helpful and it could improve my life because I didn’t know that and now I do, so its obviously gonna improve the way I obviously have to exercise better, to eat better, things like that and go for regular testing. Because other then that, I didn’t know too much about it (03 AB Caucasian aged 36)  I was really curious to know about it and now I am let’s say, a little bit more informed; what is it, how to prevent it, because this is something maybe that could come back in other pregnancies. I don’t know in the future what will be, so I need to take care of myself from now and I need to make sure that I do everything it requires to prevent any heart problem in the future So when we talked about the signs because in the pregnancy I had the signs but I didn’t know like the shortness of breath, the dizziness and the extreme fatigue and everything. In my mind I was just thinking it was the; like the pregnancy but I didn’t know that it was related to something more serious. So I think that having the QPL and asking the questions to the doctor helped me put the finger on the right words or the right reason it happened to me (13 QC African aged 42)  Oh [QPL] did [help] a lot. I find that helped a lot with this is just the eye-opening portion of it. The educative part, letting me know that this is your risk. The other part is; it’s just that… that you have the problem but it tells you how to prevent it and then after preventing it, it also tells you should it; the prevention… work and you end up developing it. This is what you have to do kind of thing also helps. So it’s kind of like an educative tool that helps you to re-examine certain aspect of your condition also. That’s what I found was very helpful about the whole thing to think about future development of it, this really, really helped me open my eyes to know that okay, you know what? You are not out of the woods yet, there’s a possibility, so you have to take care of yourself and you have to do this. And then even knowing the preventing heart disease aspect also was very, very helpful. And then the signs of heart disease, I found the answer were very, very good. It helped me to also look at things. I’ve always thought okay, heart disease you can have but then to have them down, like this makes it very easy to remember and then think about them when it happens (14 ON African aged 46)  It definitely gave me a more in depth kind of idea of how and like why this is something that happens in women who have had issues with the high blood pressure in pregnancy. I definitely feel like I know a lot more and I kind of know the warning signs and I know how to prevent it, well compared to like my last pregnancy. Like it was just; I knew it but I didn’t know any details. I didn’t know how to prevent it and I didn’t know why it would happen (23 ON Caucasian aged 30) | It helped me to understand some other things to prevent heart disease. It helped me to understand some of the risks. It helped me to think about why I got high blood pressure in my pregnancy in the first place and what else I need to do to help prevent heart disease or be monitored more closely. It helped me think about all of that stuff more than I was prior to this (06 ON Caucasian aged 36)  If I don’t have this QPL with me, if my friend, she didn’t give, send this QPL to me, so maybe I have; I’m still having a fake idea about the whole concept... Like I was; like before I have; I was having some curiosity about my health issues, that why my; I am checking my BP on a regular basis it is good but during the pregnancy it was bad. So, I have a very vague kind of scenario about the high BP in my mind but as QPL is in front of me, as there is something to read, something I have so that I can elaborate my mind towards the high BP issue so that I can search more about the things. Otherwise, maybe if I’m a layman I don’t have a medical background or things like that. I can have a vague kind of idea about high BP. Maybe I will have some misconception of the things like that. So with the QPL it is more, I can rely more on the; what my doctor is saying because I can understand that now better because with the QPL even I went, when I went to the doctor I searched little about those things on the Google specifically what is there in the QPL. What is the basic thing I need to be aware of as a layman (07 ON South Asian aged 40)  Yah, because as I mentioned that if I am; I also want to know the answers of the questions which are in the QPL, so I search it on the Google and it’s helpful for; in my future. That what are the symptoms, what are the main causes of this, like HDP, so it’s easy, like you can, you can know about the main [cause] or you can, said that the core importance of all the core reasons or the symptoms of HDP and then I search it and then I know that what’s the main symptoms and how it affect us, all those things. So, yah it’s beneficial for me (08 ON South Asian aged 31)  It was able to help me to even like have more knowledge and have more deeper understanding like of the questions. Just basically learning about the symptoms, the various ways of preventing the HDP, the medications, how to better care for ourselves and also like, as us women I felt it was for me, educative (09 ON African aged 32)  I think it was a good refresher of what of the stuff I need to take to prevent risk, increased risk of heart disease. It was actually; I appreciated having the appointment with my healthcare provider because its I have a lipid specialist whose following me right now and he does most of the follow-up with me with regards to my plan of care or plan of action for my own heart. So it was nice to sit down with somebody I know a little bit more personally to have a discussion and make sure that they’re also on the same page as the lipid specialist and that we could discuss any concerns or and go over plan of action moving forward. She also reminded me that the signs and symptoms of heart disease and heart… in women is different than a man and so, to… what plans and symptoms to lookout for (15 ON South Asian aged 41)  Yah because based on the conversation, I feel like I was more enlightened and yah, I got to have more insight on some of the questions that I felt like I didn’t know how to go about, so yah. I feel like for me because I had gestational hypertension. I actually didn’t quite understand because during my child birth, I was sort of like from the conversation that I had with my friend they usually say like, oh women who have had high blood pressure they are of having heart diseases for me I didn’t quite understand the link between the two. So yah, when I had this conversation with my doctor was able to like expand on that. And yah, it actually quite made a lot of things for me because yah, it was funny that I felt like I just need to ask so that I can have a clear understanding of that, so yah (18 ON African aged 29)  I became too cautious about whatever I, about my diet first, about the being physically fit and I tried to avoid anything that might cause a stress to me, yah. Yah so it was. There are so many things I never knew that can harm me, can cause maybe the heart disorders and the pressure about food, about not having enough sleep and not watching my weight. I didn’t know that all that can cause any harm (20 QC African aged 32) |

IMPROVEMENTS: How could we improve the QPL?

Suggestion for Content

| Theme | Recruited by physicians | Recruited in other ways |
| --- | --- | --- |
| No changes needed | I personally don’t think there was really a way to improve the QPL. The questions are really good and it helps formulate that discussion between the physician and a patient (01 AB Caucasian aged 37)  No [suggestion for question] these are great questions (02 ON Caucasian aged 45)  No, I thought the format was good the way that it was set up. I couldn’t really think of anything else. I do like the fact that it was before the appointment. But other than that, I don’t. I couldn’t think of any improvements, no. I thought it was well done. The questions were clear it was easy to read (03 AB Caucasian aged 36)  No the instructions are pretty good and the questions were good… questions that were people would want to know (04 ON Caucasian aged 40)  I think like for the most part it’s very like well organized. Like I like that its kind of divided into like the 4 categories to keep it simple and then each category only has two questions. I think if it was any, like more than that it would be a little bit overwhelming. So I think like I like the layout and the simplicity of it, that it’s not like super overwhelming. I also like the resources are; like I think really important, and I do think like having it in advance is like super helpful. So I honestly don’t know how it could be improved (05 ON Caucasian aged 35)  No, I don’t have any like additions. I thought it was straight forward to use, the format was very clear. Yah, I don’t have any suggestions (23 ON Caucasian aged 30) | No, I am satisfied with QPL because it is very easy to understand. This QPL which is right now in my hand. It is, I think it is; it has covered all the things which I should know yah (07 ON South Asian aged 40)  The questions are really good. Like it’s like this are clearly mentioned that what’s the symptoms, what’s the reason, all those things that what relating to the study is really clear. So I think the basic study which you guys more like conducted, it covered all the basic study structure. So I think it’s really clear and easy to understand yah (08 ON South Asian aged 31)  No I don’t have any [suggestion]. It’s okay (10 AB South Asian aged 46)  For now because I am new to it, I don’t know in the future if I will realize there are more that you guys could add into the format, everything. For now I am really satisfied with it. For myself, I had the right questions. I have the answers and then as I said, I didn’t know about it. So I know more now. For me, it’s okay (13 QC African aged 42)  I think it’s very straightforward. The questions are very clear. It’s very simple and… so somebody who was not as knowledgeable with regards to heart disease and HDP they’d be able to pose the questions easily and see the answers easily (15 ON South Asian aged 41)  I don’t think so. For now I don’t think I do have anything to add onto that because for me, I felt, I just feel like it was all okay for me so I don’t think I do have anything to add onto that (18 ON African aged 29)  I think your presentation is quite good because most of them are not too shareable but yours is actually very readable and I very presentable, so I have no questions on that (19 ON African aged 30) |

Suggestion for format

| Theme | Recruited by physicians | Recruited in other ways |
| --- | --- | --- |
| Multiple modifiable formats to suit those who want a checklist, or to add questions or answers | I don’t need space to write stuff down personally. So maybe just like the questions because I can take notes on my phone if I could like, just like take these because you can copy and paste them but the way it’s in a box it’s a little bit different (02 ON Caucasian aged 45)  I don’t have a printer at home, so if it was a fillable form that would have been useful for me (22 ON Indigenous aged 26) | I mean if it only can be one sheet, maybe to have a place for extra questions that maybe don’t fall into these pre-set questions (06 ON Caucasian aged 36)  Yah it is easy if it is in the hard copy because I’m not that person who is having [printer]. I know I can show QPL through my mobile but for me it is easier to have the QPL as a hard copy and then I can show to the doctor if I have something in my mind I can put a note over there and I can work out for that note and later on also (07 ON South Asian aged 40)  I would think for some people if they could sort of download it on their phone and then write on it, on their phone that would probably be helpful... like a file that you know sometimes there’s forms you can fill-out on-line, so maybe if there was a link to a web-page or something and you could fill it out for yourself on-line as you were sitting there and then you would have access to that information on your phone in the future (21 QC Caucasian aged 51) |
| Enhance graphic appeal |  | For me, I feel maybe like, maybe a picture, a diagram because… help. Maybe of, like a pregnant woman and also maybe like explaining, like if you have HDP, like what you can do and yah (09 ON African aged 32)  Make it more appealing or like have, have some graphics and so it doesn’t look like a boring brochure, like it’s interesting to look at. Maybe like images, like an image of a woman and like just colors, like welcoming colors and yah, so it’s not looking like a very technical document, it looks like something you would want to peek and read (16 ON African aged 29) |

How can we help women like you use the QPL? Suggestion for dissemination

| Theme | Recruited by physicians | Recruited in other ways |
| --- | --- | --- |
| Ensure physicians are familiar with the QPL | Having the physician prepared to have the discussion and answer the questions prior to the patient coming. Sometimes physicians don’t have enough time to prepare for a patient appointment but if a physician is ready and a patient is ready to have that discussion that would be extremely helpful. So having the physician go through the QPL prior to seeing the patient would be helpful (01 AB Caucasian aged 37)  Maybe if the doctor had it, they’d be more; they would have thought about it in advance and maybe they would have answered it. I don’t know (02 ON Caucasian aged 45)  I guess this is just like a trial, so actually putting it into use to women who actually do have HDP. And doctors mentioning it to people that have HPB to go through the questionnaire. I thought this the way it was emailed to me it was good. Like if the doctor would have emailed me like in a scenario, doctor emailing it to me is good. The doctor tells me about it is good (03 AB Caucasian aged 36)  Having paper copies available at the appointment in case they forget because most of them would have probably had babies and have mom brain (04 ON Caucasian aged 40)  When he’s giving it you, he’s already looked at it in advance and he knows what they should be telling you about to get your… but if you take it there during the appointment it’s almost like they are in a rush, they’re just going to squeeze through it instead of actually taking their time to look at it and explain things to you better, yah (14 ON African aged 46)  I think that, like doctors should probably have it as well and kind of use it as like a foundation for appointments for new patients especially (22 ON Indigenous aged 26) | If I have QPL or if I have such thing earlier with me or if the gynecologist itself, suggest me, these things earlier. So maybe at that time also if I have some questions in my mind which I skipped to ask my doctor at that time, maybe then, then it, like now I can say I have more. I am more confident and I have cleared so many things about high BP and heart disease. So if it is during that period itself, through the gynecologist maybe that will be more beneficial (07 ON South Asian aged 40)  You can send it to the doctors and then… said to the doctor that you ask from your, like from your patients. Because it’s really hard for the patients to ask those questions to the doctors because some of them are not know us, like properly because we hardly visit to the family doctors most of the time if there is not emergency. So they didn’t know us, they didn’t know you guys; that you, maybe they know you guys but they didn’t, like in show interest to answer those questions. So the basic portion, the doctor portion, you can ask, give to the doctors and then they ask from the patients. So it’s easier for you (08 ON South Asian aged 31)  I feel like just creating more awareness and also getting in contact with the doctors because most doctors having contact with many women as they go for clinics and all that. Like in case you’re not able to create awareness to more women, you can contact the hospital, then get in touch with the doctors who can help in getting the QPL reach the women (17 ON African aged 30)  For reaching out to doctors and letting, them know about it. I think that would really help. And you know the doctor is reached out in a very… it means that even the patients get maybe whatever they need from the doctor because doctors are the ones who advise you on the health so for me, I was advised, it reaches out to more doctors or to; okay for us, who have already gone to the doctor, they already know about it (19 ON African aged 30 ) |
| Reach pregnant women through clinics and pre-natal classes | Anytime we have a chance especially with pregnant women or post-partum women; any, anytime we have a chance to give it to them we should give it to them. Because we never know what could happen. We never know. I would say, if we; if you know there are clinics that receiving pregnant women, I think that we should disseminate the QPL like early, may be at the end of the pregnancy, mid-pregnancy. Any doctor’s appointment, anytime. I think so (13 QC African aged 42) | You see, most of us usually meet the hospitals and you get out of 10 women, 6 of them have no idea of what’s happening or what’s going to happen and how they can… So I feel that regularly you should be visiting the hospitals and talk to the doctors on how you can reach us because for reasons I go to the hospital right now, and I have no idea what’s happening to me. And then I go back home, nobody tells me about it. Most of… actually have no like internet or even funds to get on how to get these… or how you going to get some of these questions. So I feel you should reach out a lot to the hospitals and ask about the women’s program, yah (19 ON African aged 30)  Instead of just say just emailing them, you know it’s not everyone who got access to media. Maybe you can try some seminars, you visit health facilities, you just advise the pregnant women from the facilities, the ones who are; or you just book, okay. You can give a date to a particular health facility and you know they usually have; okay, they usually have records of the ladies who go the clinics when they are pregnant and they can inform them this particular day we’ll be having a seminar, it will teach you the blah, blah, blah. So now you just go physically meet them (20 QC African aged 32) |

When is it best for women to receive the QPL (sent to women before appointments/given to women by doctors during appointments)?

| Theme | Recruited by physicians | Recruited in other ways |
| --- | --- | --- |
| In advance of appointment gives patient time to prepare so they are better able to absorb information during appointment | If you give the QPL during the appointment you might not have enough time because physicians are also very busy and a lot of the times the physician appointment times, if you have just the one issue, it’s usually restricted to 15 minutes and might not be enough (01 AB Caucasian aged 37)  I think it would be better to have the QPL prior to the appointment because that way it prepares the patient and gives them a little bit more autonomy to have that discussion instead of the physician kind of throwing it on them and saying, well you know you had hypertension, you have these risks, here are the questions, let’s go through them together, because it might not be enough time for the patient to absorb all the information versus if you have that information ahead of time whatever questions or follow-up questions you have related to it, you can ask the physician at that appointment (01 AB Caucasian aged 37)  I think it’s better to have it before because it kind of alerts you to things that maybe you didn’t think of. I read over the questions so that definitely made it a little bit easier going in. If the doctor would have said HDP I would have been like, what? Then I was like, oh like in seeing it on this paper. It’s like okay, well I’m kind of ready to know what that means when they say it (02 ON Caucasian aged 45)  I would think for myself, [I prefer having] it would be before the appointment. You know like if I have an upcoming appointment for this and I got to prepare for my appointment (03 AB Caucasian aged 36)  I think if they got it ahead of time before their appointment so they can review them ahead of time (04 ON Caucasian aged 40)  I think before is like a 100% better because you have the questions in advance, you can kind of look at it to understand what you’re going to maybe be asked or told or discuss and then like the resources that are on it are I think super important to review before because then you have a bit of an idea so you can understand even before the doctor discusses it with you and you make that plan together type thing (05 ON Caucasian aged 35)  I would say you could give it before because it’s not like questions that you could only answer by yes or no. You need to know, you need to have more details like how? What is it? What can you do? Things like that. And I think both. I think you can, you should give it before, before the appointment so the patient could read it and then get to; try to answer by him or herself all the questions. And then in a second time, discuss all those questions with the doctor and have like clearer answer like I had (13 QC African aged 42)  When you have it in advance and you have time to look at it, it helps a lot before going to see your doctor. Then you know the kind of questions to ask and it makes up; like you’re already aware of some of the questions so your brain kind of works around it when your doctor is talking you are able to grasp it better (14 ON African aged 46)  I think it’s good to have it in advance but because then like its hard to think in an appointment when there’s kind of that, like doctor patient dynamic. I find it hard to like I have a lot of anxiety in doctor’s appointments, so it’s hard to keep a train of thought. So having it in advance was very helpful (22 ON Indigenous aged 26)  I think that giving it to women before the appointment is likely is the best and the most helpful because it actually gives you time to think about what like what questions you want to ask and usually you have to do tests before the appointment and while you may not have the results, I think that it’s important to at least be aware of like what your results could be like from the blood work. And it kind of relives a little bit of stress (23 ON Caucasian aged 30) | It could be nice to have it given to women prior to their appointment so they have an idea of what they’re going to be discussing with their doctor when they come to the appointment. And it may give them an opportunity to do some of their own research on things prior to seeing their doctor. It gives them an opportunity to come up with their own questions that maybe the QPL helps prompt them; that they may not have thought of in the moment. If they only get it at their doctor’s office, they may not have time to think of other questions (06 ON Caucasian aged 36)  I think it is more good if I have the QPL before my appointment because on that note, I have my mind prepared that yes, I have to ask these things. So it is more good if I have the QPL before an appointment with my doctor (07 ON South Asian aged 40)  I think before is okay so that we are prepared right? If it is like because if I’m getting it earlier, so I have, I can see it, I can review it, right? I can go through it. So, that is a reason. Everything I prefer is a little bit earlier so I could prefer prepare myself. That is the reason why I’m saying that, before (10 AB South Asian aged 46)  I think in general its better for somebody to have it beforehand so they can you know look at it if they want too, read it over if they want to prepare as they wish rather than it; if it was a doctor just asking the questions. I think it would feel more impersonal. A little bit more intimidating if it was just the doctor asking these questions of the patient rather than the question; the patient having some time to prepare themselves mentally for the appointment (15 ON South Asian aged 41)  I would say from my experience, I would recommend before the appointment, just so that the woman can sort of look at the question, look at the answers and like think about them and then when they go for the appointment the questions are not like brand new; they’re not seeing them for the first time. They already know what to expect and they already know what they’re expecting from their appointment (16 ON African aged 29)  The women should be given the QPL before the appointment. Before they can go to see the doctor because I feel like those questions prepare somebody psychologically and when you go to the doctor you are able to know whatever question that you should ask, it gives you a clear idea of what you’re suppose to ask or what you’re going to get into that room. For instance, the relationship between you and your doctor, it prepares you psychologically that you can find the relationship very well or you can find a doctor who is not really interested with helping you and your problem. So it prepares us mentally and psychologically and one is able to go through the process in a better way (17 ON African aged 30)  I felt that only for me, I feel its better they just give it before the appointment. That may; actually make someone feel at ease and they’re aware of what kind of questions they’re going to be in the QPL. So I feel like, yah it should be done before (18 ON African aged 29)    It should be given before. Because, okay, one will try to research before the appointment and to have a little knowledge about all whatever she should expect from, from the doctor. So the doctor won’t have; the doctor too, won’t be having hard time to explain something to the patient (20 QC African aged 32)  I think it’s better [to have QPL] before [appointment] so that you could read it and do a little bit of research yourself with the [QPL]. I liked the fact that there are links to websites on the bottom of it, so that it just makes it easier for you to understand when you are having the conversation if you’ve already done a little bit of research yourself to understand some of the basic concepts. And then also, so that I could prepare which questions I wanted to answer, ask I should say. Because yah, I know she’s busy and so I don’t want to ask things that I don’t need to (21 QC Caucasian aged 51) |
| Having QPL provided by doctor before or during visit | I would think it would be easiest to have people use it if they’re having an appointment with their doctor’s office and their doctor’s office provides this prior to their appointment and ask them to fill it out (06 ON Caucasian aged 36)  I think just maybe like having it; even I think if it was sent out too early it; people would forget about it. But I think just like having it a part of like every appointment, like follow-up appointment if like when nurse books the appointments to kind of send it out to have it like included with the appointment; to have it with everyone. I think it would be like super important because people don’t really prep for the appointment and just go in like thinking like, oh I don’t even know what this appointment is; it’s about; oh I had a preeclampsia, so we’re gonna see how that’s going now. But I think it’s like super important to inform people, like oh, what, like they can kind of read things quickly in advance, like oh this is actually what heart disease is and then you can go to your appointment more informed (05 ON Caucasian aged 35)  I feel like if I was given that form during my appointment when I was getting all of this information, I would feel a little bit out of control at the appointment. But walking in with it, I felt like I was in control of how that appointment was going to go or at least a little bit anyway (23 ON Caucasian aged 30) | I think you should give this document to the doctors. And when like when the patients come to the doctor, they ask like; then the communication is more comfortable and more easy. Because it’s really hard for the patient to convince the doctor to answer those questions because they didn’t, like most of the time they didn’t interested it. So, if you guys send or connected with the doctors, they are more easy to convince their patients to answer it and the communication is more like comfortable and more, and like easy. Because the patient is like the patient has the confidence on their doctors because if there is a family doctor as if I take example of mine. I have a family doctor and I am connected with them like last two years. So, I am confident on my doctor and I am easy to communicate with them and discuss my past life or my past history, my medical history with them. So if she want to answer, ask questions I can easily answer them because I trust on them; trust on her. So I think it’s a good way you can, you send those questions to the doctors and then they discuss with their patients (08 ON South Asian aged 31)  I think its better during the appointment because the it would have prepared the doctor before and okay… let me say that for a patient, you go to the doctor because you have a problem. So and for the doctor, you get the doctor right when walking from. So the doctor might not have all the time of probably just going and knocking in and asking questions but for the doctor will have his own scheduled time and for the patient has all the time to be with the doctor. I hope I make that clear (19 ON African aged 30) |
