# Supplementary material for: Evaluation of a Question Prompt List About Cardiovascular Disease Risk and Prevention After Hypertensive Pregnancy: A Pilot Study
Source: Health Expect. 2024 Oct 30;27(6):e70085. doi: 10.1111/hex.70085 (PMC11522917; doi:10.1111/hex.70085)
Supplement: Supplementary file 6 — Supporting information. [file HEX-27-e70085-s002.docx]

**SUPPLEMENTARY FILE LEGEND**

Supp 1. Interview guide.doc

Supplementary File 1. Question guide for qualitative interviews with participating women

This file contains the interview guide used to explore how the Question Prompt List (QPL) helped participants or facilitated their discussions with healthcare providers.

Supp 2. Consultation Care Measure.doc

Supplementary File 2. Consultation Care Measure scores for perceived person-centred care

This file contains each participant’s responses to each items on Consultation Care Measure (CCM), which assesses perceived person-centered care. It outlines the mean scores and standard deviations for each participant's responses, illustrating a detailed view of how each participant rated their consultation experiences.

Supp 3. Self-efficacy.doc

Supplementary File 3. Self-efficacy for Managing Chronic Disease scores

This file includes responses of each participants to the Self-Efficacy for Managing Chronic Disease scale, which measures participant’s confidence in managing their chronic conditions. It includes detailed scores for each participant, including their mean scores and standard deviations.

Supp 4. Perceived self-management.doc

Supplementary File 4. Perceived Medical Condition Self-Management Scale scores

This file contains the responses of each participant to the Perceived Medical Condition Self-Management Scale that measures their perception to manage their health condition. It includes the mean scores and standard deviations for each participant’s responses.

Supp 5. Themes and quotes.doc

Supplementary File 5. Themes and quotes from interviews with participating women

This file contains all the quotes from the participant interviews related to how the QPL helped or impacted their discussions with their doctors.
